# Intranasal Administration of Insulin and IGF-1 Protects Hippocampal CA1 Neurons Against Transient Global Forebrain Ischemia by Inhibiting Autophagy, Apoptosis and Neuroinflammation: A Comparative Study

**DOI:** 10.3390/ijms27167183

**Published:** 2026-08-11

**Authors:** Irina O. Zakharova, Liubov V. Bayunova, Daria K. Avrova, Natalia F. Avrova

**Affiliations:** Sechenov Institute of Evolutionary Physiology and Biochemistry, Russian Academy of Sciences, Thorez av. 44, 194223 Saint Petersburg, Russia; zakhar@iephb.ru (I.O.Z.); bayunoval@mail.ru (L.V.B.); avrovacat@mail.ru (D.K.A.)

**Keywords:** transient forebrain ischemia, intranasal insulin, intranasal IGF-1, autophagy, apoptosis, neuronal viability

## Abstract

In rats with transient global ischemia of forebrain, the intranasal administration of 0.6 µg of IGF-1 or 0.6 IU (equal to 24 µg) of insulin 1 h before or 2 h after (but not 30 min after) the ischemic episode and then daily during 7 days of reperfusion diminished the death of neurons in CA1 region of hippocampus to the levels observed in this region of sham-operated rats. The intracerebroventricular administration of autophagy and apoptosis inhibitors (3-MA and Z-VAD-FMK, respectively) markedly increased the viability of neurons in hippocampal CA1 region, providing evidence that autophagic and apoptotic deaths of neurons predominate in this brain region of rats subjected to forebrain ischemia and reperfusion, while intranasal administration of IGF-1 and insulin are able to diminish or prevent autophagic and apoptotic death of neurons. To confirm such conclusion, we showed that intranasal administration of insulin or IGF-1 prevented the increase in the level of such autophagy markers as LCB3-II and pULK-1(Ser^555^) in the hippocampus of ischemic and reperfused brain; other data confirming the ability of IGF-1 and insulin to inhibit autophagic processes and caspase-3 activity were also obtained. IGF-1 and insulin were shown to prevent the activation of microglia and astroglia and neuroinflammation in hippocampus of ischemic and reperfused forebrain. The similarity of IGF-1 and insulin effects suggests that insulin in much higher doses than IGF-1 is able to interact not only with insulin receptors, but also with IGF-1 receptors. As oncogenic effects are characteristic of IGF-1, insulin appears to be a better candidate for use in clinics.

## 1. Introduction

Ischemic stroke is an acute cerebrovascular event characterized by the interruption in the blood supply to the brain, leading to cerebral hypoxia and tissue damage. It ranks as the second leading cause of mortality worldwide and remains a predominant contributor to long-term adult disability. The Global Burden of Disease (GBD) study—a comprehensive epidemiological framework that systematically quantifies mortality and disability attributable to major diseases, injuries, and their associated risk factors—has documented a marked increase in both the global incidence and absolute disease burden of ischemic stroke in recent years, a trend largely driven by population growth and demographic aging [1,2,3]. Furthermore, growing evidence suggests that this upward trajectory will persist globally, with the most pronounced escalation projected to occur in low-income countries [4]. The pathophysiology of stroke and other brain injuries resulting from ischemia and reperfusion involves a complex interplay of excitotoxicity, oxidative stress, inflammation, and two interrelated forms of neuronal death: apoptosis and over-activated autophagy [5,6,7,8,9]. Although apoptosis has long been recognized as a viable target for therapeutic intervention, autophagy was historically ascribed a purely protective role. However, contemporary evidence indicates that excessive autophagic activation during ischemia and reperfusion critically undermines neuronal viability in the brain [10,11,12,13]. Consequently, neuroprotective agents capable of preventing neuronal loss while concurrently modulating both apoptotic and autophagic pathways are of considerable clinical interest.

Insulin and insulin-like growth factor-1 (IGF-1) belong to such neuroprotectors. They are evolutionarily related substances belonging to the insulin family. They converge upon a shared set of intracellular signaling cascades, including activation of the PI3K-Akt pathway, inhibition of AMPK, and modulation of ERK activity [14,15,16,17,18,19]. Nevertheless, despite the common downstream effectors of insulin and IGF-1 pathways, their functional activities are characterized by both overlap and notable divergence [20,21,22,23]. A key distinction lies in their secretion dynamics: insulin is released transiently in response to nutrient intake, whereas IGF-1 is secreted constitutively. At the cellular level, IGF-1 predominantly exerts pleiotropic bioactivities, promoting growth, cell survival, and the maintenance of cellular homeostasis. Conversely, insulin regulates classic anabolic metabolism by enhancing glucose and amino acid transport, stimulating glycogen, lipid, and protein synthesis, while concurrently inhibiting lipolysis and protein degradation. Within the central nervous system, insulin’s actions extend beyond systemic metabolic regulation to directly influence brain function, wherein it exerts pronounced neuroprotective effects [24,25,26,27,28]. Disruption of insulin action in the brain not only impairs neuronal function and synaptogenesis but also modulates tau protein phosphorylation, an early pathological hallmark of Alzheimer’s disease [29]. Thus, IGF-1 predominantly mediates long-term signaling to determine cell fate and prevent neuronal death, whereas insulin primarily exerts metabolic effects, although it also possesses neuroprotective properties [29,30].

Experiments in rat embryos with knockout of the IGF-1 or insulin receptors confirm such differences in the primary functions of these two ligands in animals and humans [31,32]. Brain IGF-1 levels during ischemia and reperfusion serve as a prognostic marker, with higher concentrations predicting a more favorable outcome following ischemic injury [33]. These data identify IGF-1 as a protective agent with considerable therapeutic potential against ischemia-reperfusion injury. The cytoprotective properties of IGF-1 have been extensively investigated in animal models of stroke, including middle cerebral artery occlusion (MCAO) and other forms of cerebral ischemia-reperfusion injury [34,35,36,37,38,39,40,41,42,43]. However, substantial safety concerns have been raised regarding the potential of IGF-1 to promote benign and malignant neoplasm formation in both human recipients and experimental animal models [22,23,44]. The recommended use of IGF-1 in clinics is currently limited to cases of severe growth retardation due to significant primary IGF-I deficiency in children and adolescents [45,46,47]. The neuroprotective effects of insulin have now been demonstrated in both animal models and human studies to support its therapeutic application for cognitive impairment in neurodegenerative diseases [48,49,50,51].

In order to determine whether insulin can protect brain tissue from ischemic and reperfusion injury as effectively as IGF-1, it is important to compare their abilities to prevent the apoptotic and autophagic death of neurons caused by this pathology, using the same disease model. To date, however, no such comparative studies have been reported. At the same time, such head-to-head evaluations are critical. If insulin proves to be as effective as IGF-1 in preventing neuronal apoptosis and excessive autophagy triggered by ischemia–reperfusion, it could be recommended for use in clinics as a safe and efficient therapeutic agent.

This study has three primary objectives: (a) to characterize apoptotic and autophagic neuronal death in the hippocampal CA1 region of rats following transient global ischemia; (b) to identify and compare the intranasal doses of insulin and IGF-1 that confer similar neuroprotection; and (c) to elucidate their mechanisms by measuring caspase-3 activity (as an index of apoptosis) and autophagic marker levels (as an index of autophagy). In a complementary set of analyses, immunohistochemical (IHC) and morphological approaches will be used to evaluate the effects of ischemia-reperfusion and intranasal insulin or IGF-1 on hippocampal glial cells. Particular emphasis will be placed on microglial activation and astrogliosis, given their critical roles in driving neuroinflammation and shaping the local cytokine milieu.

## 2. Results

**Figure 1:**
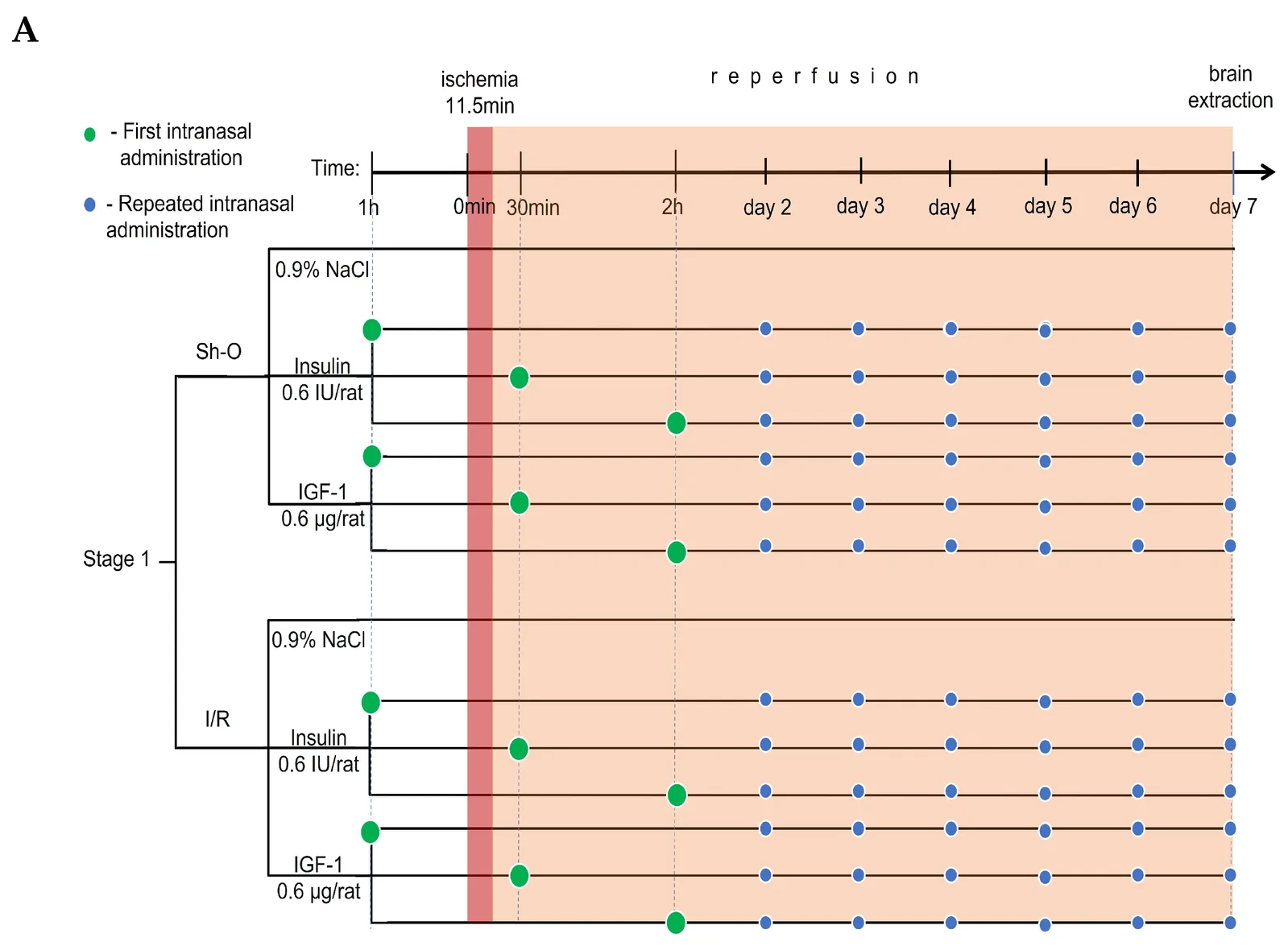

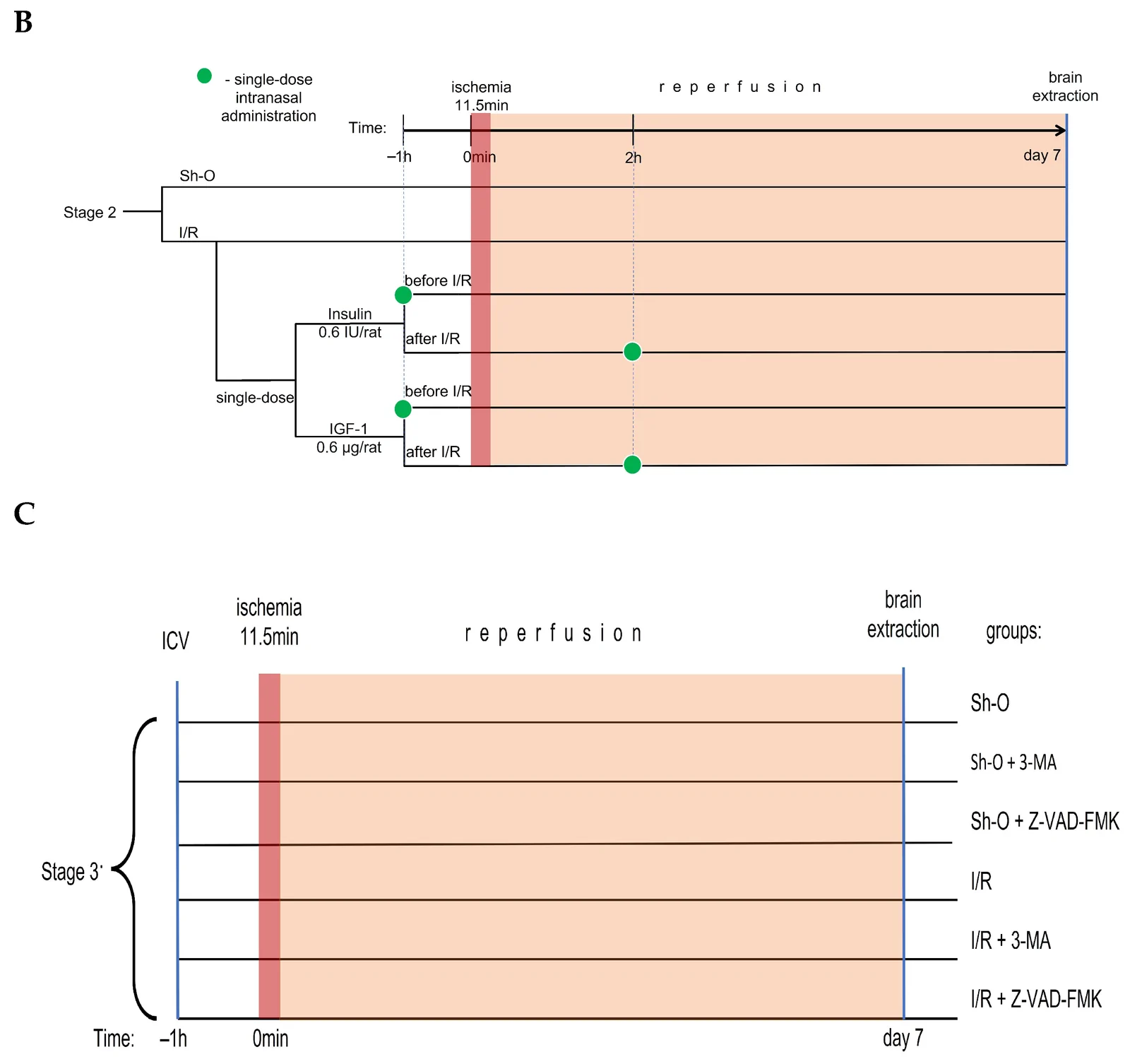
Experimental timelines illustrating the treatment sequence and animal grouping during the studies of neuronal viability in hippocampal CA1 region. (**A**) Timelines of insulin and IGF-1 intranasal multiple administrations during studies of neuronal viability in hippocampal CA1 region. (**B**) Timelines of one intranasal administration of insulin and IGF-1 during studies of neuronal viability in hippocampal CA1 region. (**C**) Timelines of intracerebroventricular administration of autophagy and apoptosis inhibitors (3-methyladenin and Z-VAD-FMK, respectively) during studies of neuronal viability in hippocampal CA1 region. Abbreviations used: Sh-O—sham-operated rats; I/R—rats subjected to ischemia and reperfusion.

This study aimed to compare the efficacy of intranasal insulin versus intranasal IGF-1 in preventing or reducing autophagic and apoptotic neuronal death in the hippocampal CA1 region of rats subjected to global forebrain ischemia induced by carotid artery occlusion combined with hypotension, followed by reperfusion. The neuroprotectors were administered at three time points for the first time: 1 h before ischemia, as well as 30 min and 2 h after ischemia, with subsequent daily administration throughout the 7-day reperfusion period (Figure 1A). Viable neurons in the hippocampal CA1 subfield were then quantified using Nissl staining.

**Figure 2:**
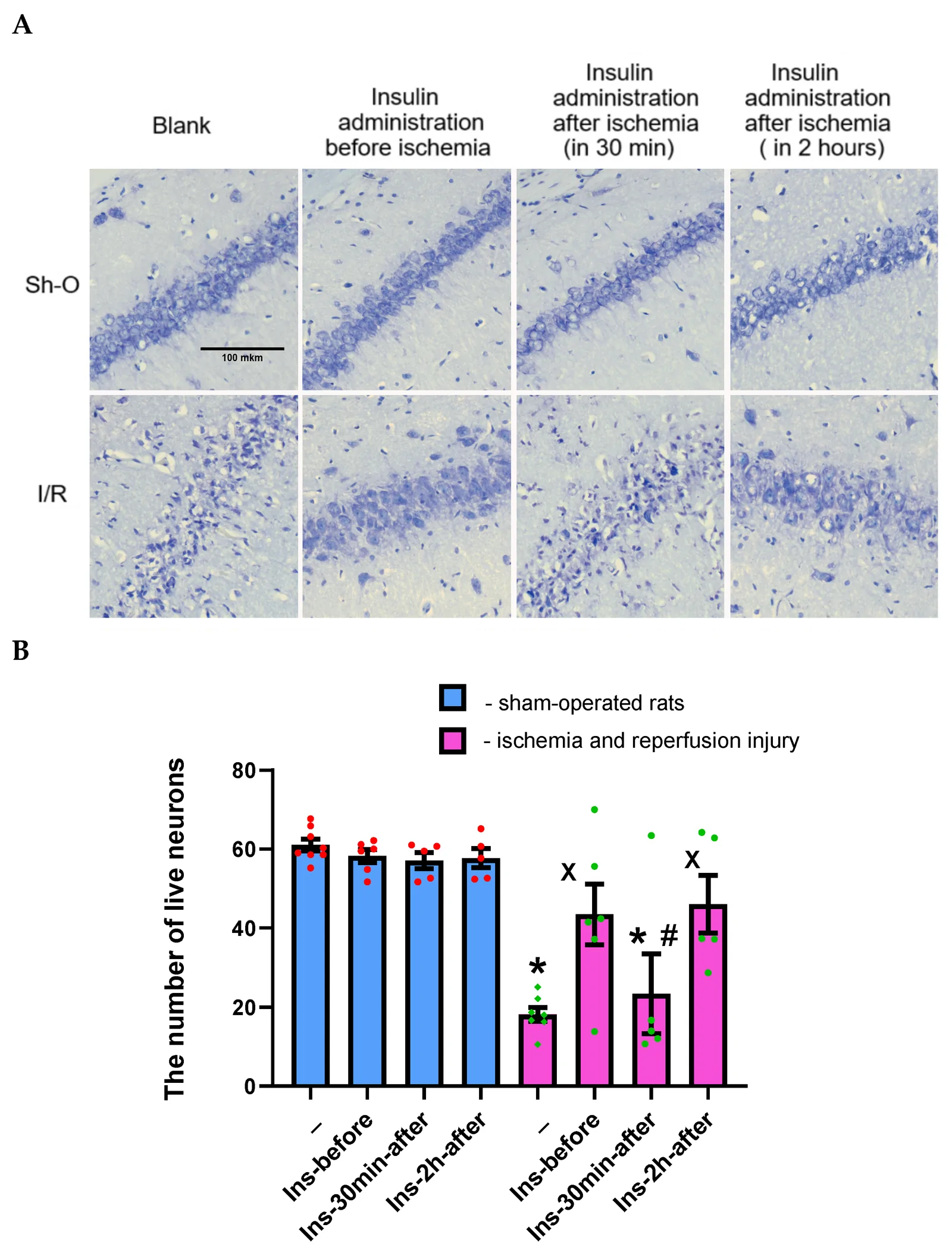
Effect of intranasal administration of insulin (0.6 IU/rat/daily) on the viability of neurons in the CA1 region of the hippocampus after global forebrain ischemia and 7-day reperfusion. Abbreviations used: Sh-O—sham-operated rats; I/R—rats subjected to ischemia and reperfusion; Ins-insulin; Ins-before—insulin was administered 1 h before the beginning of ischemic episode and then daily during 7 days of reperfusion, Ins-30min-after and Ins-2h-after—insulin was administered 30 min and 2 h after the end of ischemic episodes, respectively, and then daily during 7 days of reperfusion. (**A**) Representative photomicrographs of the CA1 region of the hippocampus after Nissl staining. Scale: 100 µm. (**B**) Quantitative analysis of live neurons in a linear 300 µm segment of the CA1 pyramidal cell layer. Data are presented as mean ± SEM (*n* = 5–6 rats per group; for each rat, at least 30 determinations were performed). Statistical significance was assessed using one-way ANOVA followed by Tukey’s post hoc test for multiple comparisons: *—as compared to sham-operated rats, *p* < 0.001; #—as compared to sham-operated rats which received administration of the same doses of insulin, *p* < 0.001; X—as compared to rats subjected to ischemia and reperfusion, *p* < 0.05.

**Figure 3:**
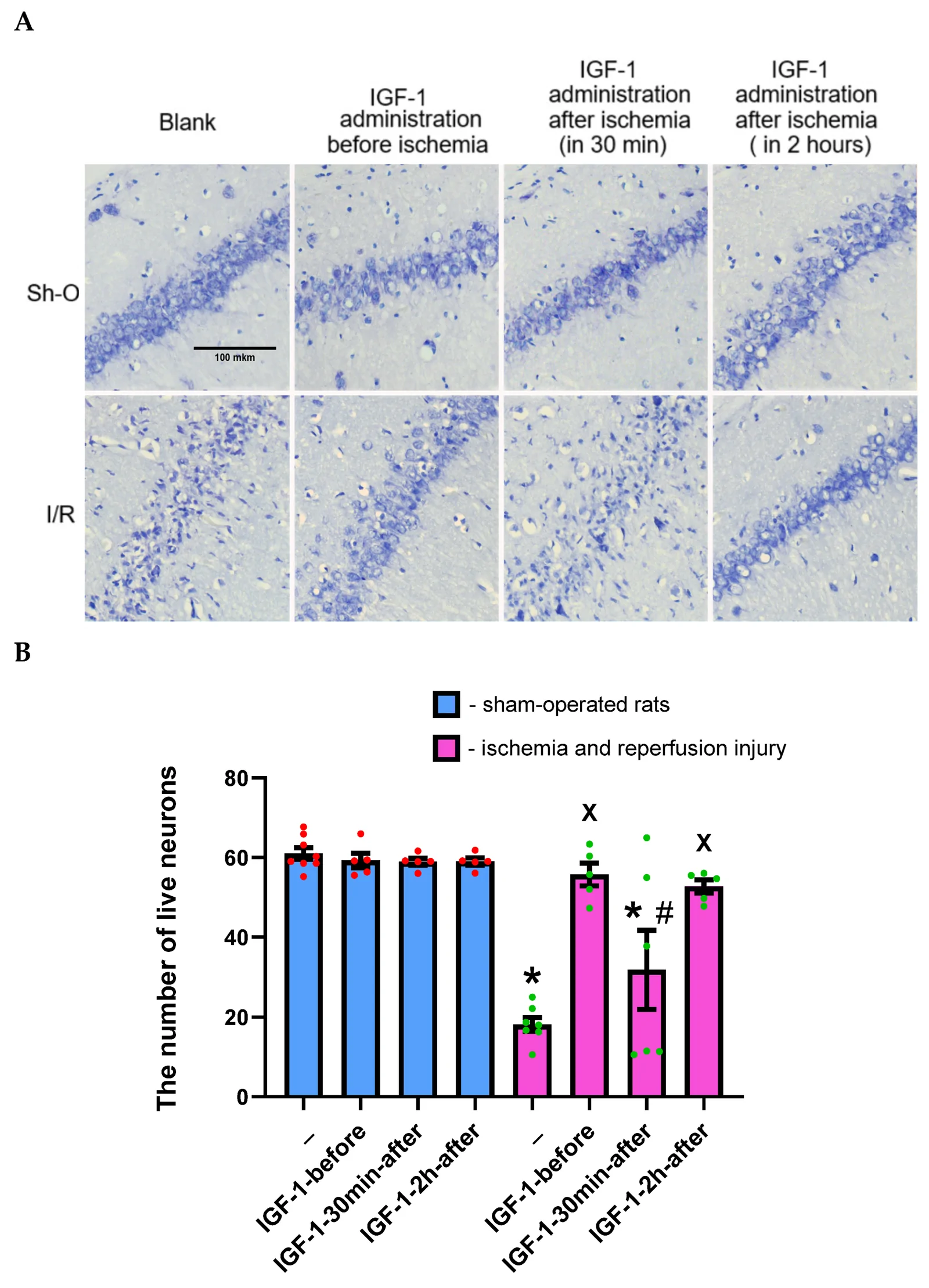
Effect of intranasal administration of insulin-like growth factor-1 (IGF-1) (0.6 µg/rat/daily) on the viability of neurons in the CA1 region of the hippocampus after global forebrain ischemia and 7-day reperfusion. Abbreviations used: Sh-O—sham-operated rats; I/R—rats subjected to ischemia and reperfusion; IGF-1-before—IGF-1 was administered 1 h before the beginning of ischemic episode and then daily during 7 days of reperfusion, IGF-1–30min-after and IGF-1–2h-after—IGF-1 was administered in 30 min and 2 h after the end of ischemic episodes, respectively, and then daily during 7 days of reperfusion. (**A**) Representative photomicrographs of the CA1 region of the hippocampus after Nissl staining. Scale: 100 µm. (**B**) Quantitative analysis of live neurons in a linear 300 µm segment of the CA1 pyramidal cell layer. Data are presented as mean ± SEM (*n* = 5–6 rats per group; for each rat, at least 30 determinations were performed). Statistical significance was assessed using one-way ANOVA followed by Tukey’s post hoc test for multiple comparisons: *—as compared to sham-operated rats, *p* < 0.001, #—as compared to sham-operated rats which received administration of the same doses of IGF-1, *p* < 0.001, X—as compared to rats subjected to ischemia and reperfusion, *p* < 0.001.

In this study, insulin (0.6 IU) and IGF-1 (0.6 µg) were administered intranasally. In the main series of experiments, the ischemia-reperfusion (I/R) group exhibited 18.2 ± 1.7 viable neurons per 300 µm in the hippocampal CA1 region. In contrast, sham-operated animals (Sh-O) displayed 61.0 ± 1.5 viable neurons per 300 µm. Thus, transient global ischemia followed by 7 days of reperfusion resulted in approximately 70% neuronal death (Figure 2 and Figure 3). As shown in Figure 2 and Figure 3, intranasal administration of either insulin or IGF-1—given once either 1 h before or 2 h after the ischemic episode, and then daily throughout the 7-day reperfusion period (Figure 1A)—increased the number of viable neurons in the CA1 region by 2- to 3-fold compared to the I/R group. A direct comparison of the two compounds revealed no significant differences in their neuroprotective efficacy at the doses tested (0.6 IU for insulin vs. 0.6 µg for IGF-1), regardless of whether they were administered 2 h after or 1 h before ischemia. Specifically, when given 2 h post-ischemia, IGF-1 increased neuronal survival to 52.7 ± 1.7 cells/300 µm (I/R-IGF-1−2 h-after group; *p* < 0.001 vs. I/R), while insulin increased survival to 46.0 ± 7.3 neurons/300 µm (I/R-Ins-2h-after group; *p* < 0.05 vs. I/R). No statistically significant differences were detected between the two treatment groups under any administration schedule (Figure 2 and Figure 3). In contrast, no protective effect was observed when the first intranasal administration of either compound was performed 30 min after the onset of ischemia–reperfusion, followed by daily doses over the 7-day reperfusion period (Figure 2 and Figure 3). This lack of effect may be attributed to the inability of insulin and IGF-1 to exert neuroprotection during the acute phase immediately following reperfusion onset. Under this protocol, the second administration occurred approximately 24 h later—well after the closure of the therapeutic window. Given the absence of a positive effect with the 30 min post-ischemia administration, further experiments were conducted to explore the therapeutic window by administering the drugs intranasally 1 h before or 2 h after the ischemic episode. Collectively, these findings underscore that the timing of the first administration is critical for the neuroprotective action of both intranasal insulin and IGF-1, as it significantly influences their efficacy.

We also investigated the role of daily insulin and IGF-1 administration during 7 days of reperfusion in the neuroprotective effect of these substances. As shown in Table 1, a single dose of insulin (Figure 1B) given either 1 h before or 2 h after the ischemic episode did not significantly increase the number of viable neurons in the hippocampal CA1 region, indicating no protective effect. Similarly, a single dose of IGF-1 administered 2 h after ischemia failed to provide significant neuroprotection. However, when IGF-1 was given once, 1 h before the ischemic episode, it significantly improved neuronal viability in the CA1 region, although its effect was weaker than when this single pre-ischemic dose was followed by daily IGF-1 administration throughout the 7-day reperfusion period (*p* < 0.05). These findings demonstrate that both the timing of the initial dose (0.6 IU of insulin or 0.6 µg of IGF-1 per animal) and the continued daily administration during reperfusion are critical for achieving pronounced neuroprotection in a rat model of transient global forebrain ischemia (Figure 2 and Figure 3, Table 1). Notably, no significant difference in the neuroprotective efficacy was observed between insulin and IGF-1.

**Figure 4:**
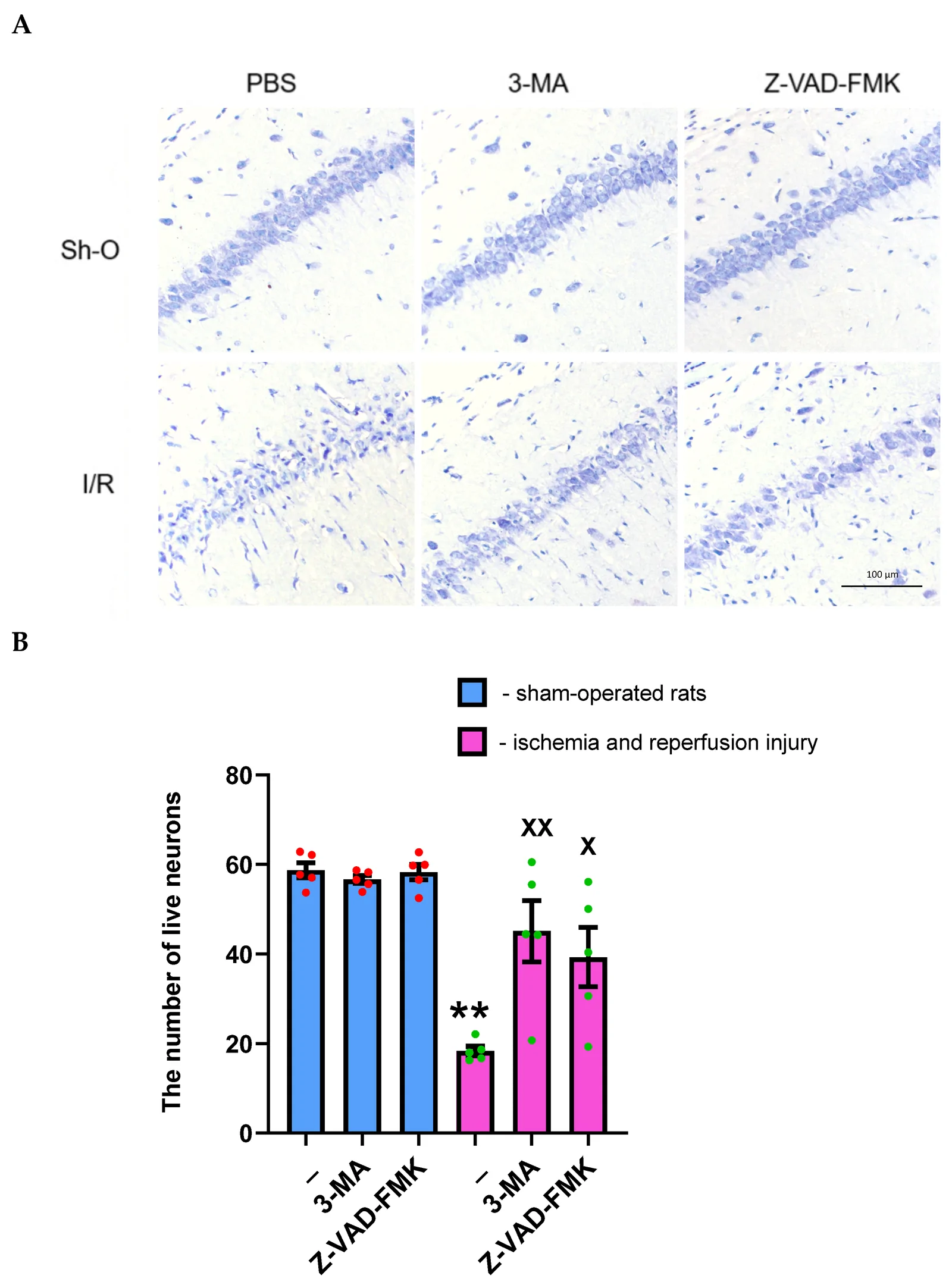
Effect of intracerebroventricular administration of 3-MA and Z-VAD-FMK on the viability of neurons in the CA1 region of the hippocampus after global forebrain ischemia and 7-day reperfusion. Inhibitors of autophagy and apoptosis (3-MA and Z-VAD-FMK, respectively) were administered to rats 1 h before the beginning of ischemic episode. Abbreviations used: Sh-O—sham-operated rats; I/R—rats subjected to ischemia and reperfusion; PBS—phosphate-buffered saline, 3-MA—3-methyladenine, Z-VAD-FMK—carbobenzoxy-valyl-alanyl-aspartyl-[O-methyl]-fluoromethylketone. (**A**) Representative photomicrographs of the CA1 region of the hippocampus after Nissl staining. Scale: 100 µm. (**B**) Quantitative analysis of live neurons in a linear 300 µm segment of the CA1 pyramidal cell layer. Data are presented as mean ± SEM (*n* = 5 rats per group; for each rat, at least 30 determinations were performed). Statistical significance was assessed using one-way ANOVA followed by Tukey’s post hoc test for multiple comparisons: **—as compared to sham-operated rats, *p* < 0.001, X and XX—as compared to rats subjected to ischemia and reperfusion, X—*p* < 0.05, XX—*p* < 0.01.

To assess the relative contributions of apoptosis and excessive autophagy to neuronal death in the CA1 region of the hippocampus following global forebrain ischemia and 7 days of reperfusion, we administered the autophagy inhibitor 3-methyladenine (3-MA) or the pan-caspase inhibitor carbobenzoxy-valyl-alanyl-aspartyl-[O-methyl]-fluoromethylketone (Z-VAD-FMK) via intracerebroventricular (ICV) injection (Figure 1C). In rats subjected to ischemia–reperfusion, ICV delivery of 3-MA significantly increased the number of viable neurons—defined as those with regularly shaped, round cell bodies—in the CA1 region from 18.37 ± 1.04 to 45.10 ± 6.85 neurons per 300 µm length (*p* < 0.01, Figure 4). The increase in surviving neurons following autophagy inhibition (approximately 26.73 neurons per 300 µm) is interpreted as the number of neurons that would otherwise have died as a result of autophagic cell death. This value represents more than 50% of the total neuronal loss attributable to ischemia–reperfusion injury under the experimental conditions used in this study.

Apoptosis also contributed substantially to overall neuronal death following ischemia and reperfusion. ICV administration of the pan-caspase inhibitor Z-VAD-FMK significantly increased the number of viable neurons in the CA1 region from 18.37 ± 1.04 to 39.31 ± 6.61 neurons per 300 µm (*p* < 0.05, Figure 4). The difference between these values (approximately 20.94 neurons per 300 µm) represents the estimated number of neurons lost specifically to apoptosis under the present experimental conditions. The difference between the number of neurons rescued by 3-MA (autophagy inhibition) and that rescued by Z-VAD-FMK (apoptosis inhibition) was not statistically significant. Our results clearly show that the excessive activation of autophagy and apoptosis contributes to neuronal death in hippocampal CA1 region caused by two-vessel occlusion of the carotid arteries with hypotension and subsequent forebrain reperfusion. The results obtained suggest that autophagy and apoptosis belong to the main mechanisms responsible for neuronal death in this brain region.

**Figure 5:**
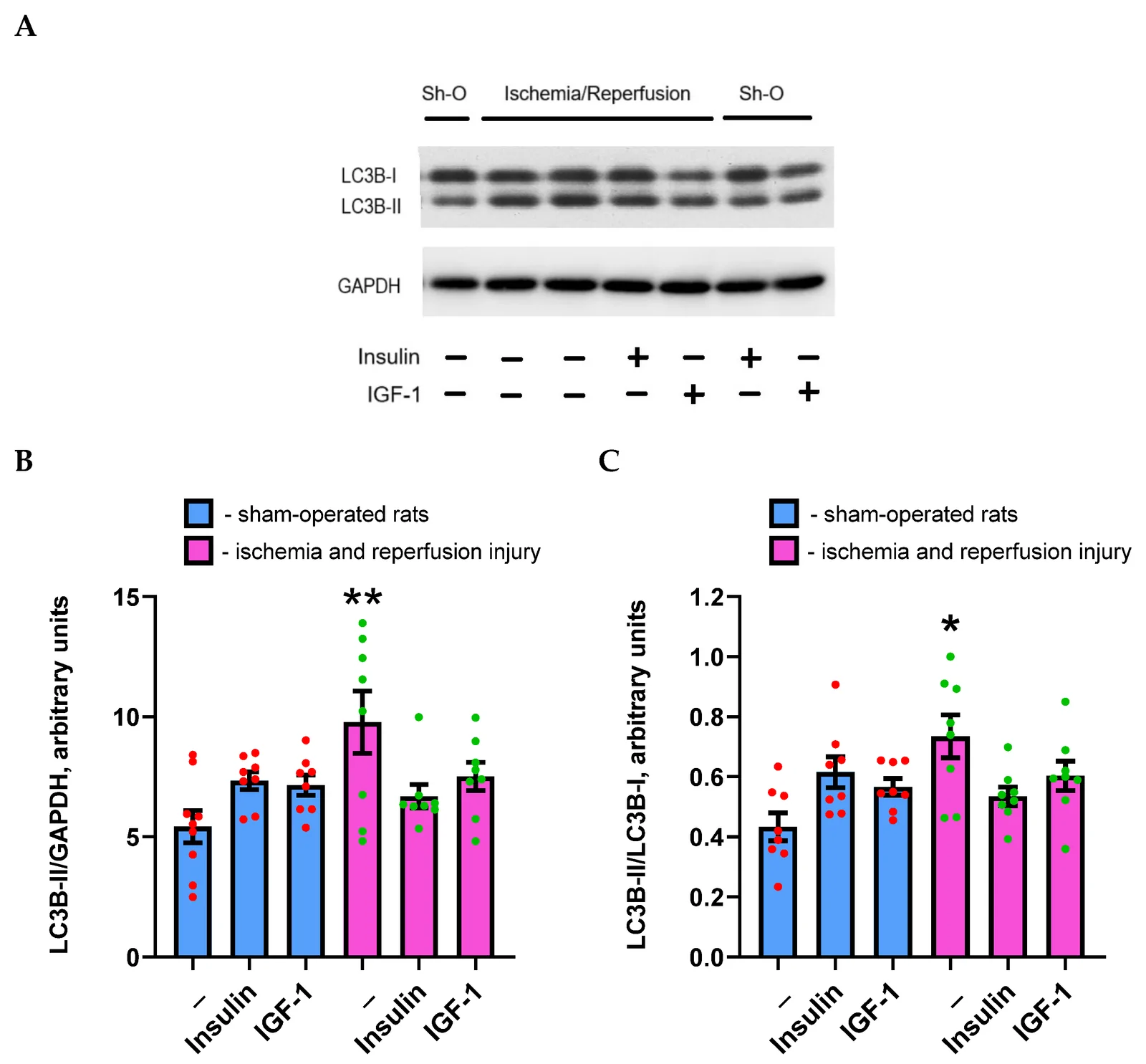
Effect of global forebrain ischemia and intranasal administration of insulin (0.6 IU/rat) and IGF-1 (0.6 μg/rat) on LC3B-II expression in the hippocampus of the rat brain after 1 day of reperfusion. Abbreviations used: Sh-O—sham-operated rats. (**A**) Representative immunoblots. (**B**) Results of the LC3B-II/GAPDH ratio in the hippocampus of the rat brain. (**C**) Results of the LC3B-II/LC3B-I ratio in the hippocampus of the rat brain. Data are presented as mean ± SEM (*n* = 6–8). Statistical significance was assessed using one-way ANOVA followed by Tukey’s post hoc test for multiple comparisons: * and **—as compared to sham-operated rats, *—*p* < 0.05, **—*p* < 0.01.

Having established that excessive autophagy contributes substantially to neuronal death after 7 days of reperfusion, we next investigated whether intranasal administration of insulin or IGF-1) could inhibit autophagy in the rat hippocampus, and we assessed their effects on the autophagy-related markers LC3B-II and phosphorylated ULK-1 at Ser^555^ (pULK-1(Ser^555^)). Immunoblotting analysis revealed that one day of reperfusion following ischemia significantly activated autophagic processes, as evidenced by increased ratios of LC3B-II to GAPDH (Figure 5B) and LC3B-II to LC3B-I (Figure 5C), indicating elevated levels of the autophagosome-associated marker LC3B-II. Importantly, intranasal administration of either insulin or IGF-1 prevented the increase in both ratios, thereby blocking the ischemia–reperfusion-induced activation of autophagy (Figure 5). These results suggest that insulin and IGF-1 may act as negative regulators of autophagy in the hippocampus under these pathological conditions.

**Figure 6:**
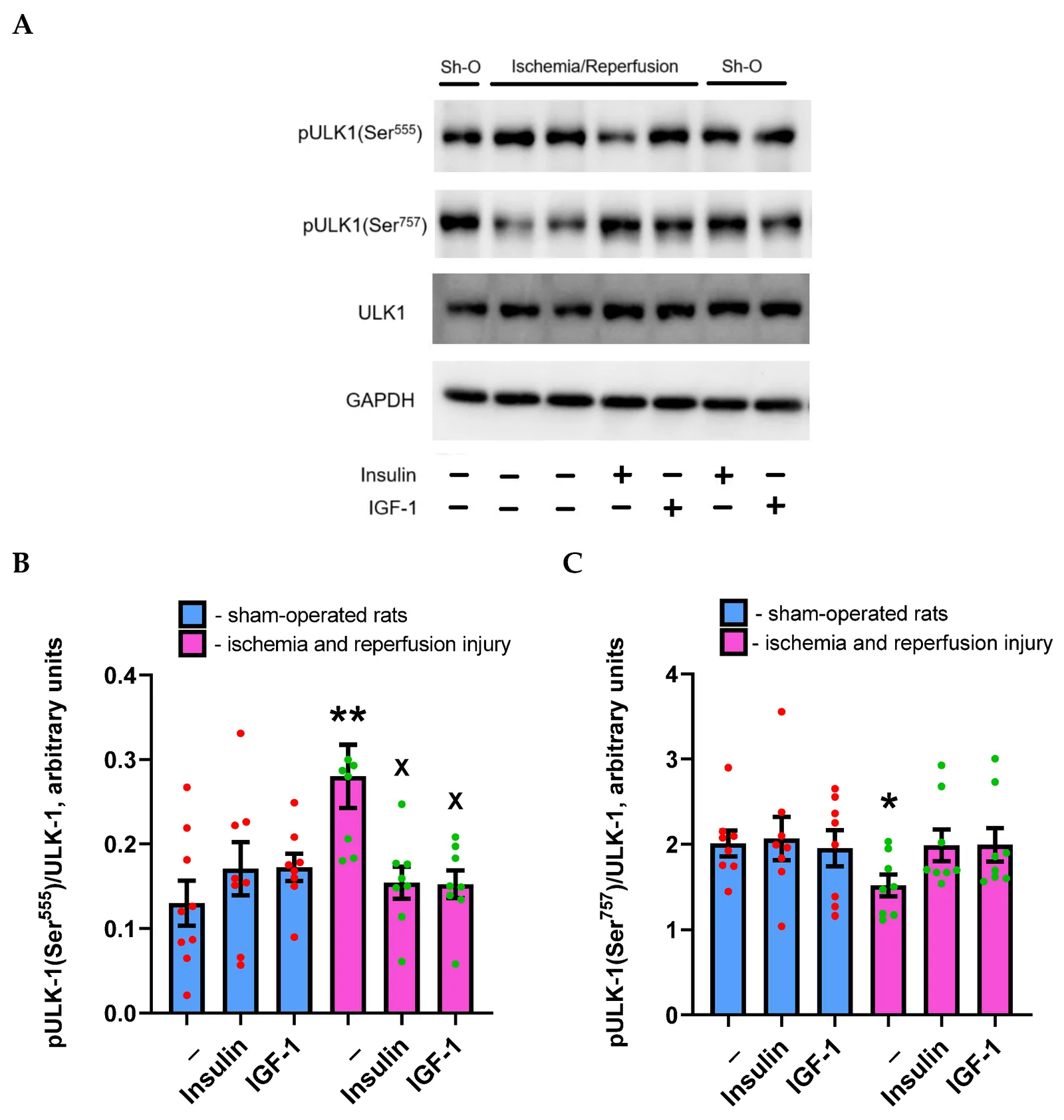
Effect of global forebrain ischemia and intranasal administration of insulin (0.6 IU/rat) and IGF-1 (0.6 μg/rat) on the phosphorylation level of protein kinase ULK-1 at Ser^555^ and Ser^757^ in the hippocampus of the rat brain after 1 day of reperfusion. Abbreviations used: Sh-O—sham-operated rats. (**A**) Representative immunoblots. (**B**) Results of the ULK-1(Ser^555^)/ULK-1 ratio in the hippocampus of the rat brain. (**C**) Results of the ULK-1(Ser^757^)/ULK-1 ratio in the hippocampus of the rat brain. Data are presented as mean ± SEM (*n* = 6–8). Statistical significance was assessed using Student’s *t*-test: * and **—as compared to sham-operated rats, *—*p* < 0.05, **—*p* < 0.01; X—as compared to rats subjected to ischemia and reperfusion, *p* < 0.05.

The ability of intranasal insulin or IGF-1 to prevent autophagy activation was further confirmed by assessing the phosphorylation status of ULK-1, a key component of the autophagy initiation complex. Phosphorylation of ULK-1 at Ser^555^ activates the enzyme, whereas phosphorylation at Ser^757^ inhibits its activity. As shown in Figure 6, ischemia followed by 1 day of reperfusion significantly increased the pULK-1(Ser^555^)/ULK-1 ratio (*p* < 0.01) and decreased the pULK-1(Ser^757^)/ULK-1 ratio (*p* < 0.05) in the hippocampus, indicating robust activation of autophagy. However, intranasal administration of either insulin or IGF-1 one hour before ischemia significantly reduced the pULK-1(Ser^555^)/ULK-1 ratio to levels close to those in sham-operated animals, thereby preventing autophagy activation. Consistently, the decrease in pULK-1(Ser^757^) levels observed after ischemia–reperfusion was no longer detectable in rats pretreated with insulin or IGF-1 (Figure 6C).

**Figure 7:**
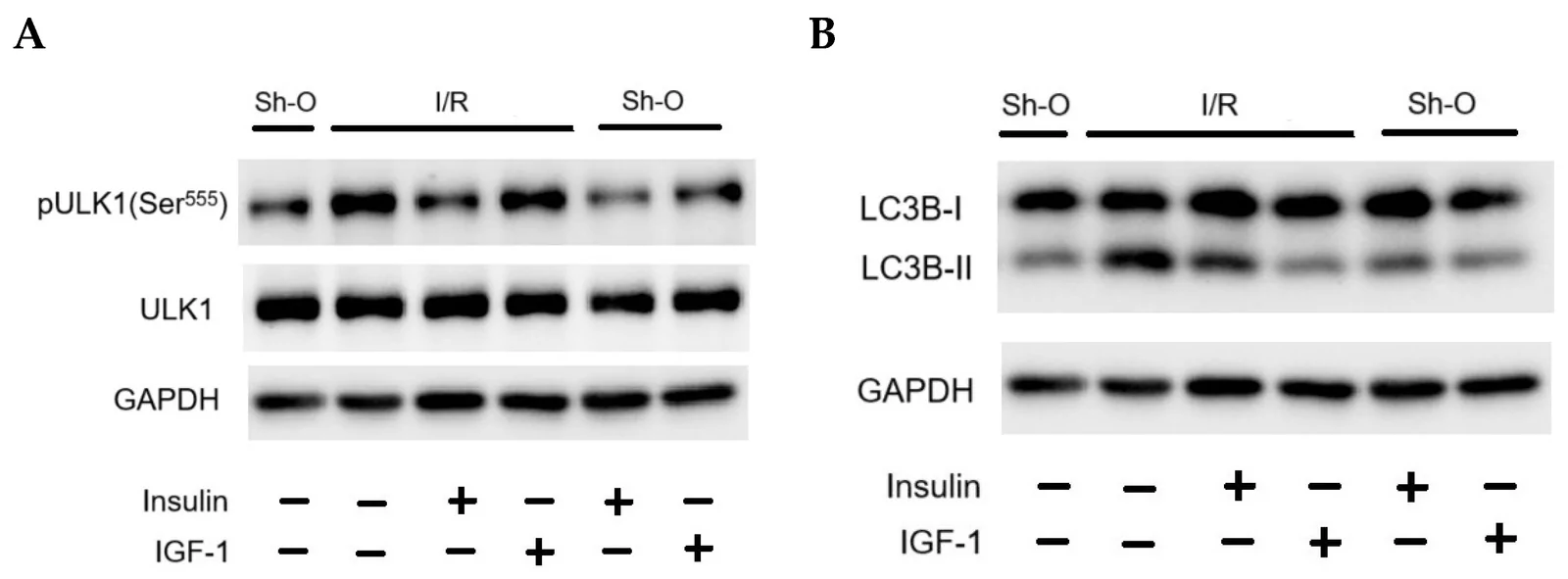

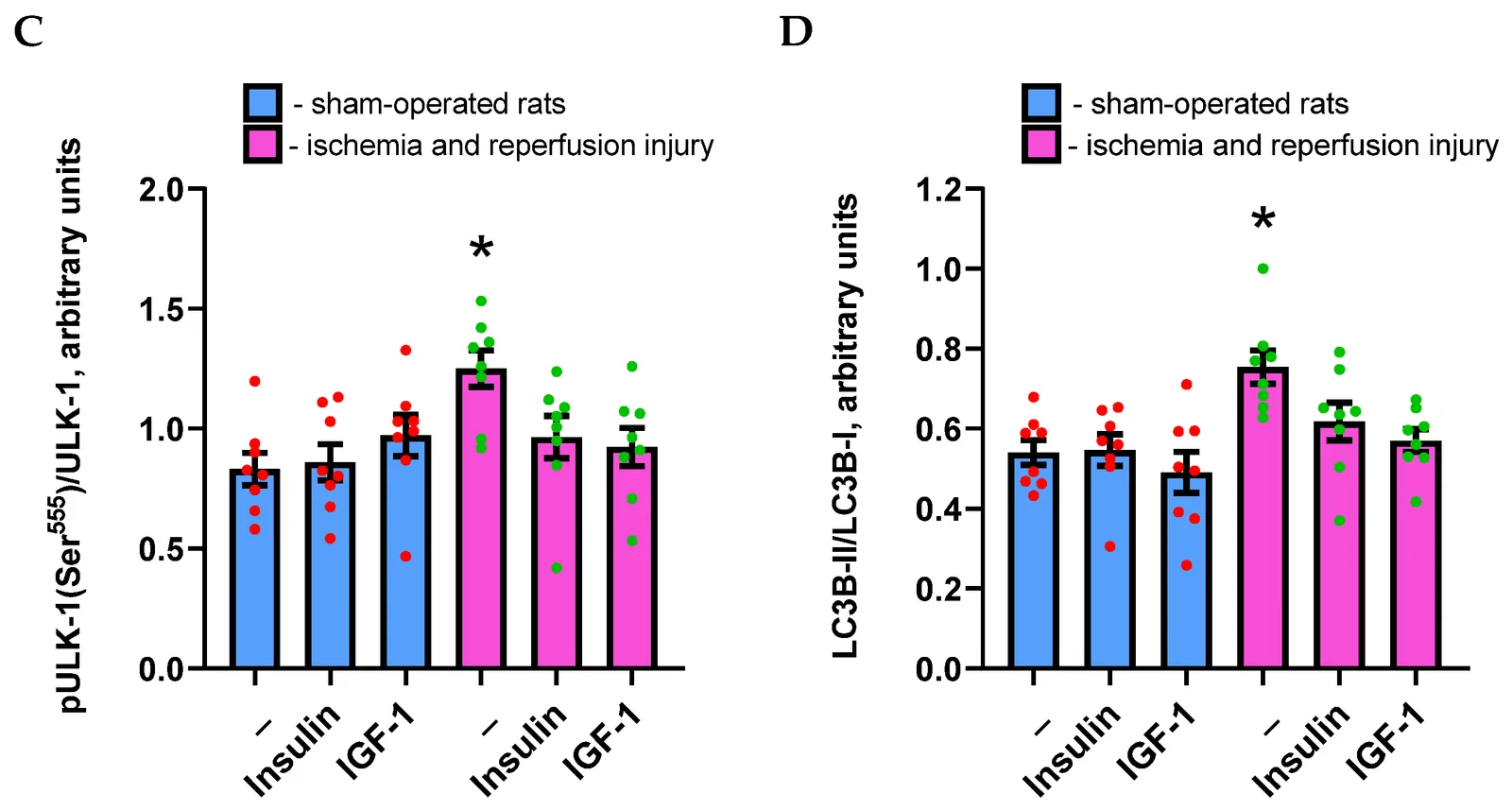
Effect of global forebrain ischemia and intranasal administration of insulin (0.6 IU/rat) and IGF-1 (0.6 μg/rat) on the phosphorylation level of protein kinase ULK-1 at Ser^555^ and expression of LC3B-II in the hippocampus of the rat brain after 7 days of reperfusion. Abbreviations used: Sh-O—sham-operated rats; I/R—rats subjected to ischemia and reperfusion. (**A**,**B**) Representative immunoblots. (**C**) Results of the ULK-1(Ser^555^)/ULK-1 ratio in the hippocampus of the rat brain. (**D**) Results of the LC3B-II/LC3B-I ratio in the hippocampus of the rat brain. Data are presented as mean ± SEM (*n* = 6–8). Statistical significance was assessed using one-way ANOVA followed by Tukey’s post hoc test for multiple comparisons: *—as compared to sham-operated rats, *—*p* < 0.05.

Autophagy remained activated in hippocampus even after 7 days of reperfusion, as evidenced by elevated pULK-1(Ser^555^) levels compared with sham-operated controls (Figure 7A,C). Importantly, intranasal administration of insulin or IGF-1 given before ischemia and then daily throughout the 7-day reperfusion period prevented this sustained increase, effectively inhibiting autophagy over the longer term. The level of another marker of autophagy (LC3B-II) was also shown to increase in the hippocampus of ischemic and reperfused for 7 days forebrain (Figure 7B,D). But intranasal administration of insulin or IGF-1 1 h before ischemia and then daily during the 7-day reperfusion period prevented this increase. These data confirm the ability of insulin and IGF-1 to inhibit the activation of autophagy caused by global ischemia and reperfusion.

**Figure 8:**
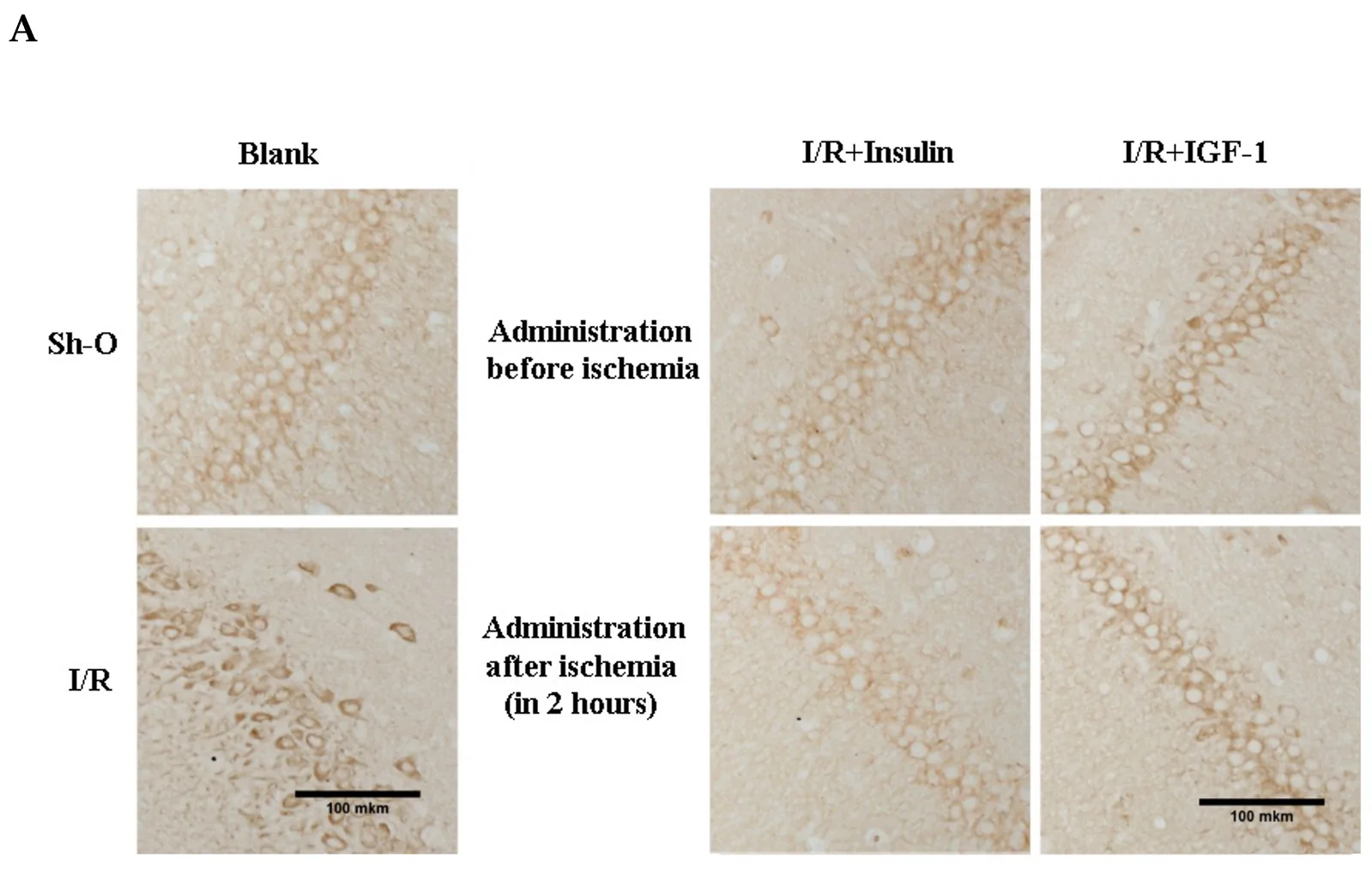

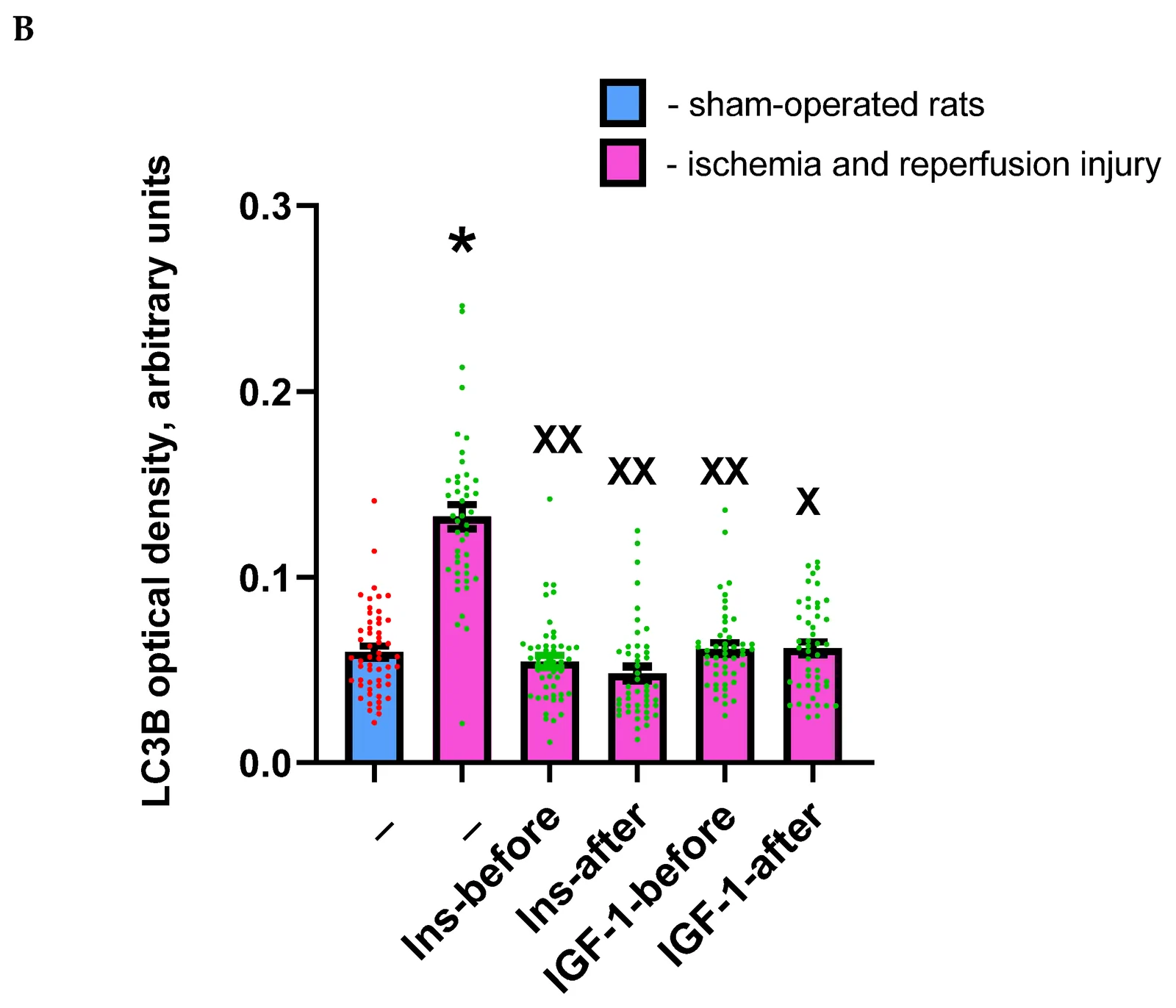
Effect of global forebrain ischemia and intranasal administration of insulin (0.6 IU/rat) and IGF-1 (0.6 µg/rat) on the expression of LC3B in the CA1 pyramidal neurons of the hippocampus after 7 days of reperfusion. Abbreviations used: Sh-O—sham-operated rats; I/R—rats subjected to ischemia and reperfusion; Ins-before and IGF-1-before—insulin or IGF-1 was administered 1 h before the beginning of ischemic episode and then daily during 7 days of reperfusion; Ins-after and IGF-1-after—insulin or IGF-1 was administered 2 h after the end of ischemic episode and then daily during 7 days of reperfusion. (**A**) Representative photomicrographs of the CA1 region of the hippocampus after IHC reaction with antibodies to LC3B, having the greatest affinity to LC3B-II. Scale: 100 µm. (**B**) Optical density of LC3B-immunopositive cells. Data are presented as mean ± SEM (*n* = 3 rats per group; for each rat, at least 15 determinations were performed). Statistical significance was assessed using one-way ANOVA followed by Tukey’s post hoc test for multiple comparisons: *—as compared to sham-operated rats, *p* < 0.001, X and XX—as compared to rats subjected to ischemia and reperfusion, X—*p* < 0.01, XX—*p* < 0.001.

To further assess autophagic activity in surviving pyramidal neurons of the CA1 hippocampal region after 7 days of reperfusion, we performed immunohistochemistry (IHC) using an antibody specific for LC3B (Cell Signaling Tech., Danvers, MA, USA), which exhibits high sensitivity for the autophagosome-associated marker LC3B-II (Figure 8). In the ischemia–reperfusion (I/R) group, IHC revealed a significant increase in the LC3B immunoreactivity in the CA1 region compared with sham-operated controls, confirming persistent autophagy activation.

Intranasal administration of insulin or IGF-1, given either 1 h before ischemia or 2 h after the onset of reperfusion and then daily throughout the 7-day reperfusion period, reduced the LC3B immunoreactivity to control levels observed in sham-operated animals. These findings indicate that both insulin and IGF-1 effectively inhibit autophagy activation in the CA1 hippocampus even when administered after the ischemic insult.

**Figure 9:**
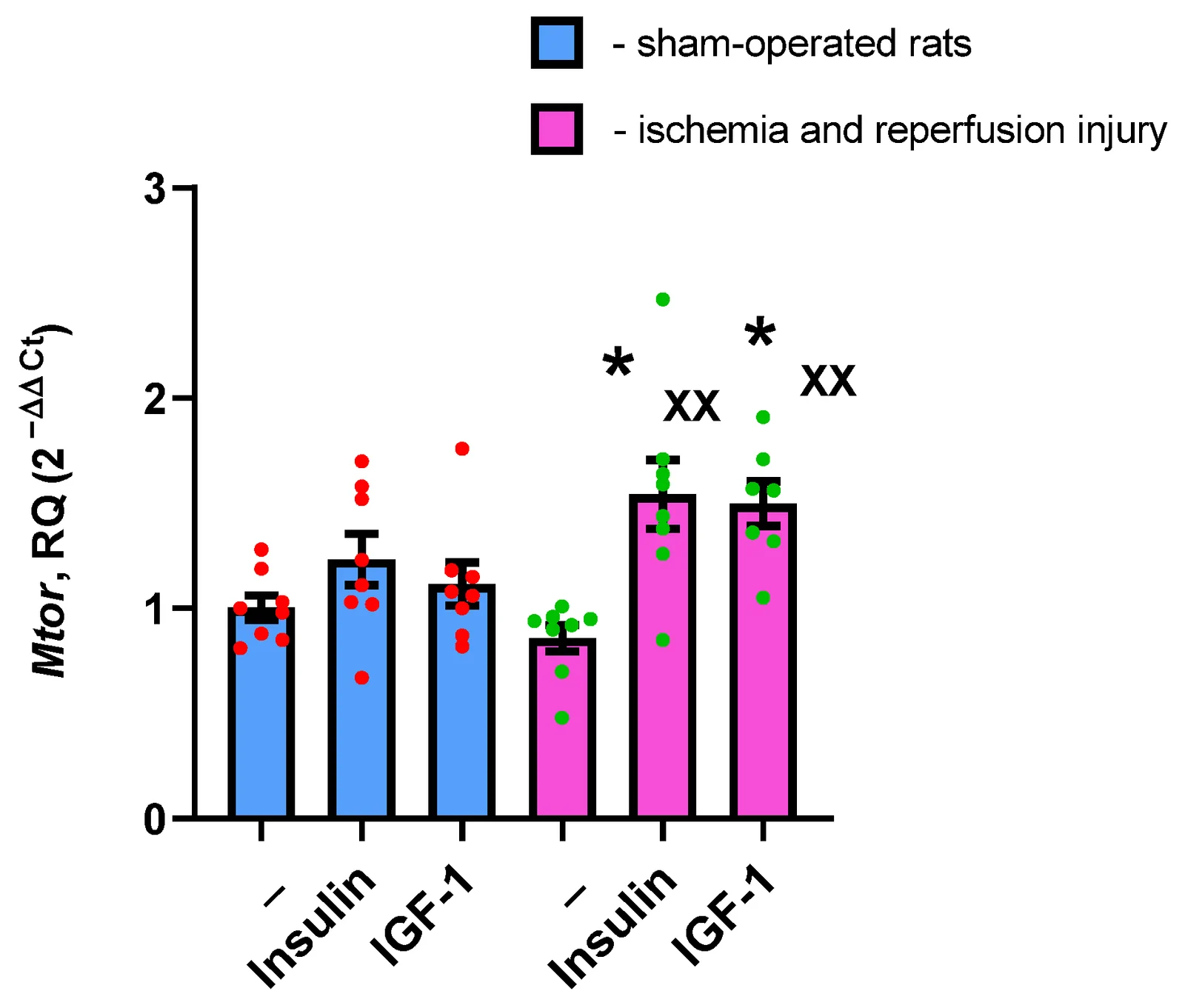
Effect of global forebrain ischemia and intranasal administration of insulin (0.6 IU/rat) and IGF-1 (0.6 µg/rat) on the expression of the *Mtor* gene in the hippocampus. Insulin and IGF-1 were administered 1 h before the ischemic episode and then daily during 7 days of reperfusion. Results are presented as mean ± SEM (*n* = 8 animals/group). Statistical significance was assessed using one-way ANOVA followed by Tukey’s post hoc test for multiple comparisons: *—compared to sham-operated rats, *p* < 0.05; XX—as compared to rats subjected to ischemia and reperfusion, XX—*p* < 0.01.

Further supporting the ability of insulin and IGF-1 to inhibit autophagy, real-time PCR analysis revealed that intranasal administration of either peptide significantly increased the expression of the *Mtor* gene—a negative regulator of autophagy—in the hippocampus of rats subjected to global forebrain ischemia and 7 days of reperfusion (Figure 9). No significant differences were observed between the effects of insulin and IGF-1 in sham-operated animals; neither substance affected *Mtor* gene expression (Figure 9). These results provide complementary evidence at the transcriptional level that insulin and IGF-1 suppress autophagy, at least in part, through upregulation of mTOR.

**Figure 10:**
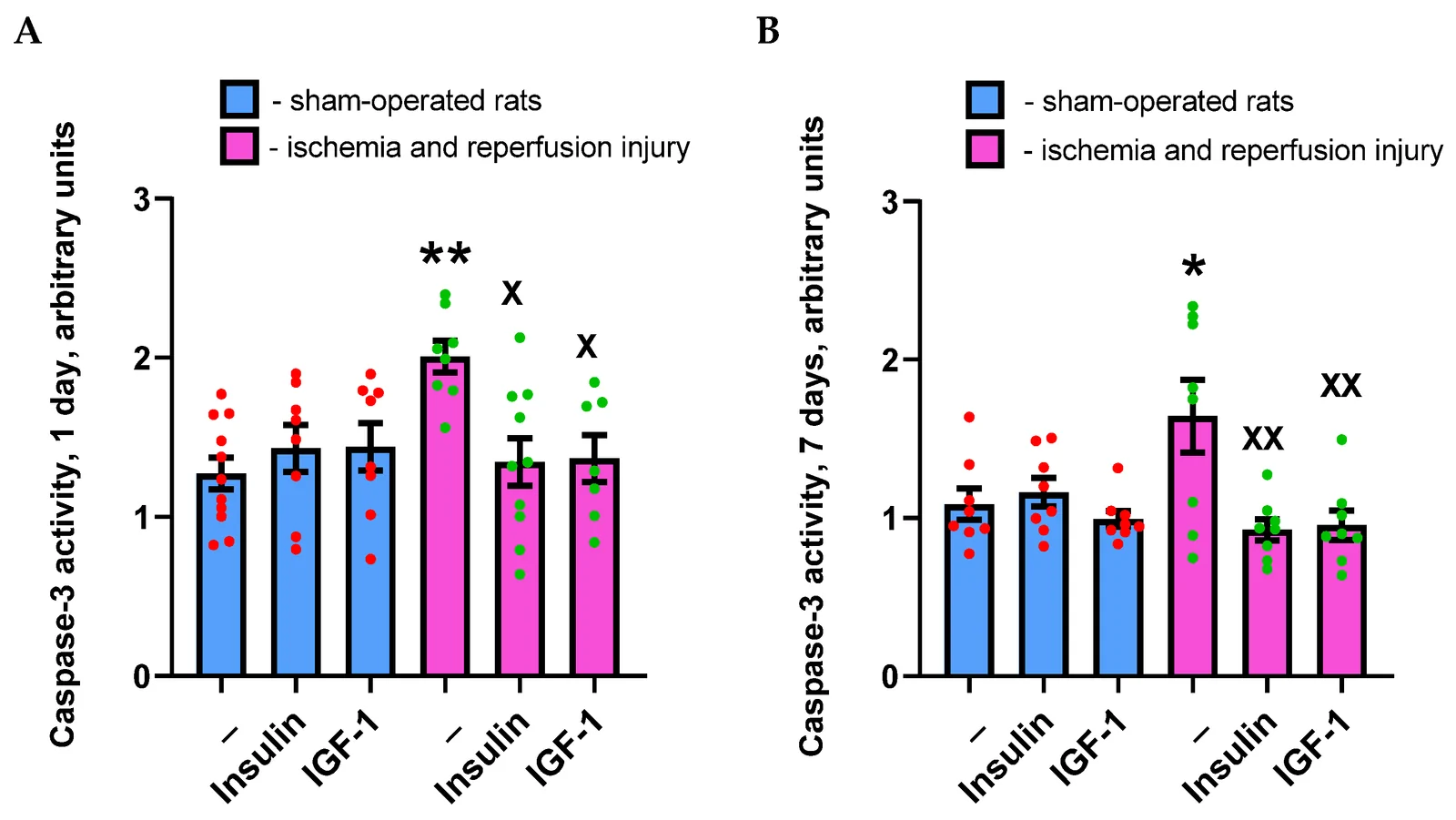
Effect of global forebrain ischemia and reperfusion and intranasal administration of insulin (0.6 IU/rat) and IGF-1 (0.6 µg/rat) on caspase activity in the hippocampus after 1 and 7 days of reperfusion. (**A**) The data on caspase activity in the hippocampus of the rat brain after 1 day of reperfusion. (**B**) The data on caspase activity in the hippocampus of the rat brain after 7 days of reperfusion. Data are presented as mean ± SEM (*n* = 8–10). Statistical significance was assessed using one-way ANOVA followed by Tukey’s post hoc test for multiple comparisons: * and **—as compared to sham-operated rats, *—*p* < 0.05, **—*p* < 0.01; X and XX—as compared to rats subjected to ischemia and reperfusion, X—*p* < 0.05, XX—*p* < 0.01.

In addition to inhibiting autophagy, insulin and IGF-1 also exerted a marked antiapoptotic effect, as demonstrated by changes in caspase-3 activity (Figure 10A,B). Global forebrain ischemia followed by 1 or 7 days of reperfusion significantly increased caspase-3 activity in the hippocampus. However, intranasal administration of insulin (0.6 IU/rat) or IGF-1 (0.6 µg/rat) 1 h before ischemia (and then daily in the case of 7-day reperfusion) markedly reduced caspase-3 activity to levels close to those observed in sham-operated controls. These results indicate that, similar to their inhibitory effect on autophagy, both peptides effectively suppress ischemia–reperfusion-induced apoptosis in the hippocampus.

Besides exerting anti-autophagic and anti-apoptotic effects, insulin and IGF-1 also reduced neuroinflammation following transient forebrain ischemia and reperfusion. Almost immediately after the onset of ischemia, microglia become activated, initiating inflammatory responses that can exacerbate neuronal injury. However, a potential beneficial role of microglia under these conditions cannot be excluded. To assess the status and number of activated microglia, we performed immunohistochemical (IHC) analysis of Iba-1 (ionized calcium-binding adapter molecule 1)-positive cells, a robust marker for microglia across various activation states (branched, activated, amoeboid, and dystrophic). Microglial activation is characterized primarily by morphological changes, including process thickening and cell body hypertrophy. IHC analysis revealed an increase in the number of Iba-1-immunopositive cells exhibiting these morphological features—typical of the pro-inflammatory M1 phenotype—in the CA1 region of the hippocampus.

**Figure 11:**
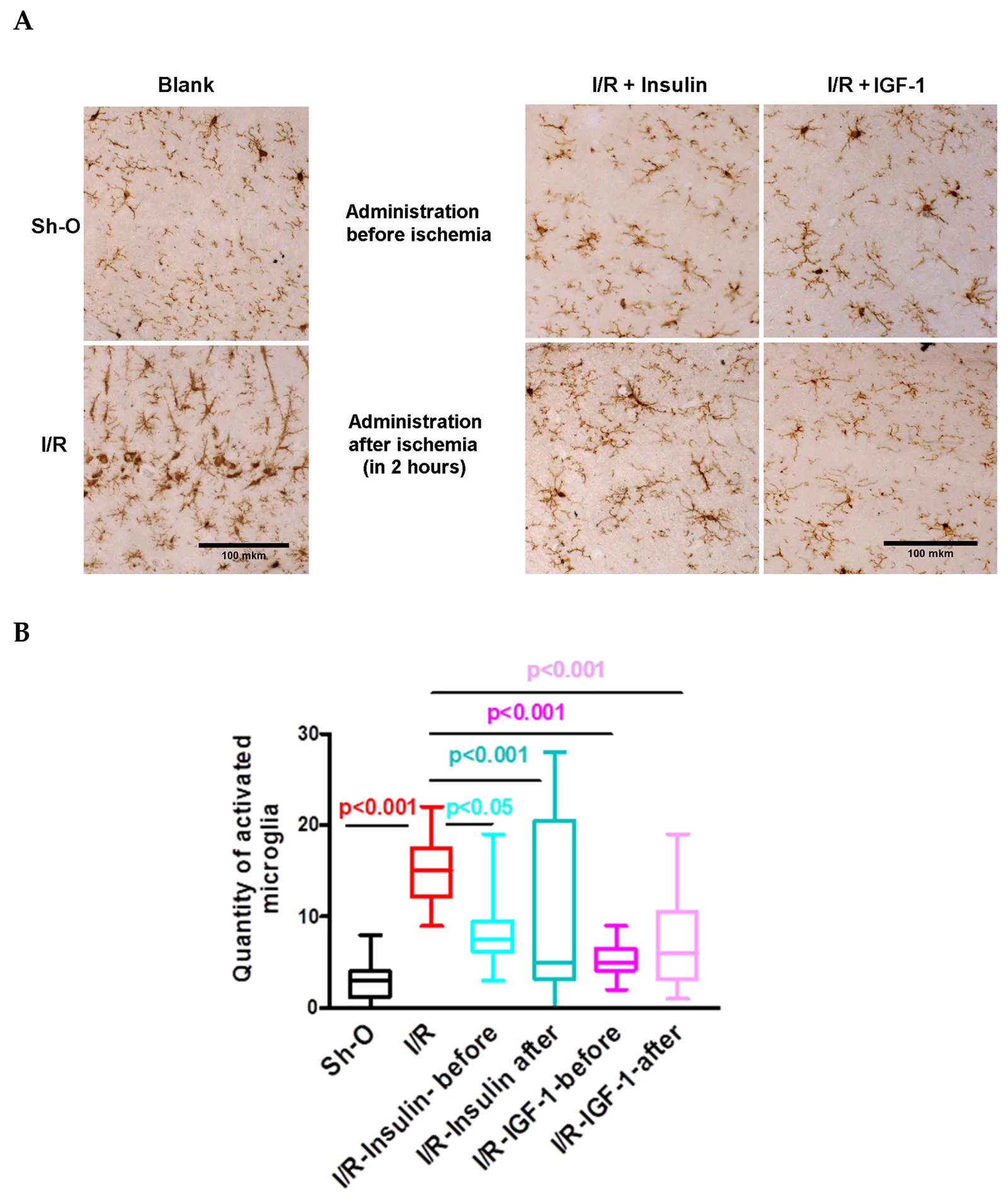
Effect of intranasal administration of insulin (0.6 IU/rat) and IGF-1 (0.6 μg/rat) on the number of activated microglia in the CA1 region of the hippocampus following global forebrain ischemia and 7 days of reperfusion. Abbreviations used: Sh-O—sham-operated rats; I/R—rats subjected to ischemia and reperfusion; I/R-Ins-before and I/R-IGF-1-before—insulin or IGF-1 was administered 1 h before the beginning of ischemic episode and then daily during 7 days of reperfusion; I/R-Ins-after and I/R-IGF-1-after—insulin or IGF-1 was administered in 2 h after the end of ischemic episode and then daily during 7 days of reperfusion. (**A**) Representative photomicrographs of the CA1 region showing Iba-1-immunopositive cells. Scale bar: 100 µm. (**B**) Quantification of activated microglial cells per unit area corresponding to 300 µm length of the hippocampus. Data are presented as medians (50% of the data) with interquartile ranges (Q1, Q3) (*n* = 3 animals per group, with at least 10 determinations per animal). Significance levels were determined using the Kruskal–Wallis test followed by post hoc analysis with Dunn’s test, and are indicated in the figure.

Because the Kolmogorov–Smirnov test indicated non-normal distribution in the I/R-Ins-after- and I/R-IGF-1-after- groups, we used the nonparametric Kruskal–Wallis test to assess significance. After 7 days of reperfusion following ischemia, the number of activated microglia in the CA1 region increased five-fold, from 3 (1; 4) cells/300 µm in the sham-operated (Sh-O) group to 15 (12; 17.5) cells/300 µm in the ischemia–reperfusion (I/R) group (*p* < 0.001). Data are presented as box plots in Figure 11.

Intranasal administration of IGF-1—given either 1 h before ischemia or 2 h after reperfusion onset and then daily throughout the 7-day reperfusion period—reduced the number of activated microglia to 5 (4; 6.5) cells/300 µm and 6 (3; 10.5) cells/300 µm, respectively (*p* < 0.001 vs. I/R group for both). Thus, IGF-1 suppresses ischemia–reperfusion-induced inflammation. Similar results were obtained with intranasal insulin administered 1 h before or 2 h after ischemia, followed by daily doses for 7 days. Insulin reduced activated microglia counts to 7.5 (6; 9.5) cells/300 µm in the I/R-Ins-before group and to 5 (3; 20.5) cells/300 µm in the I/R-Ins-after-group, compared with 15 (12; 17.5) cells/300 µm in the I/R group (Figure 11). Both reductions were statistically significant (*p* < 0.05 and *p* < 0.01, respectively). Notably, when either insulin or IGF-1 was first administered 2 h after ischemia, at least one animal in each group developed severe inflammation, accounting for the large interquartile ranges observed in those groups.

**Figure 12:**
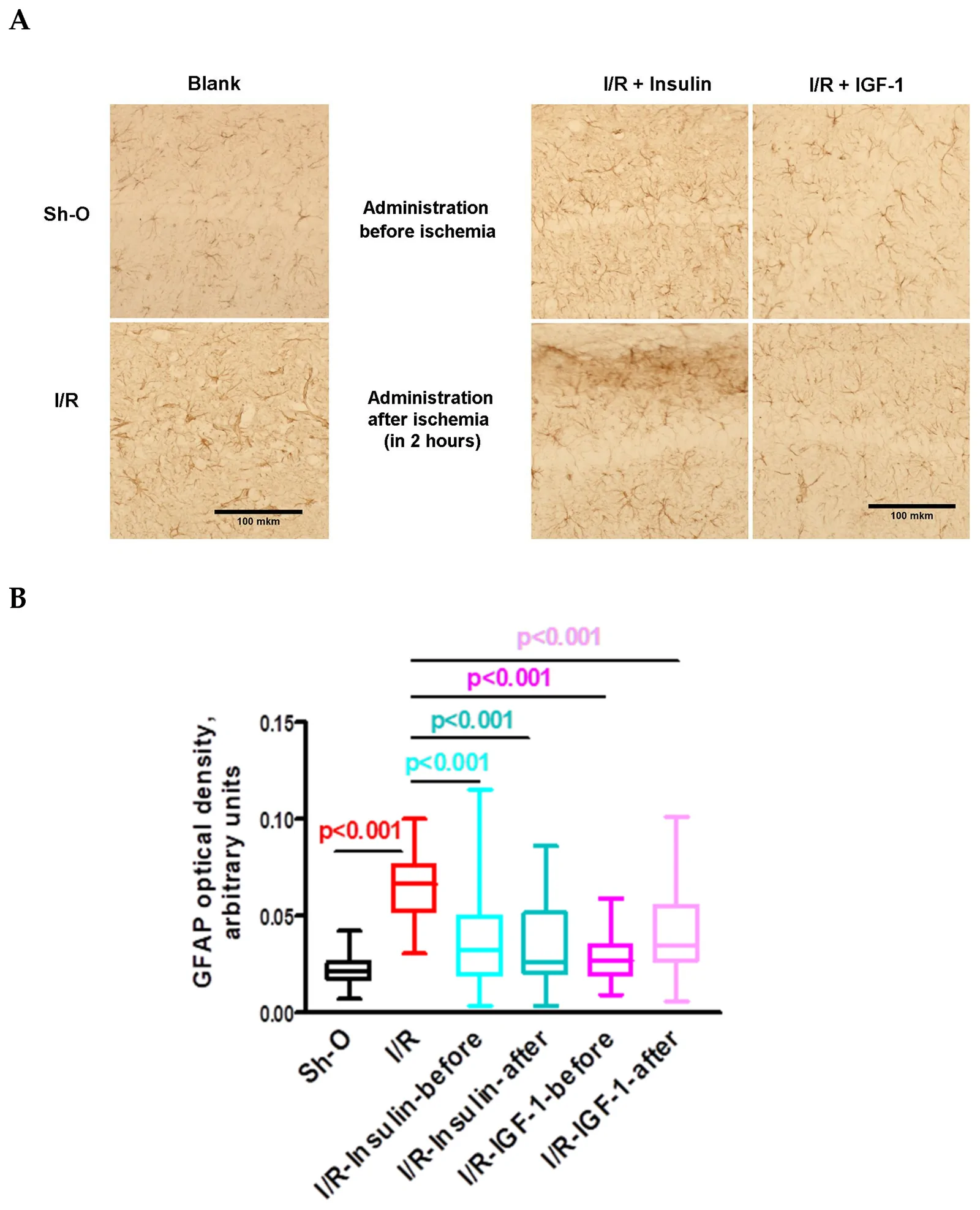
Effect of intranasal administration of insulin (0.6 IU/rat) and IGF-1 (0.6 μg/rat) on GFAP expression in the CA1 region of the hippocampus following global forebrain ischemia and 7 days of reperfusion. Abbreviations used: Sh-O—sham-operated rats; I/R—rats subjected to ischemia and reperfusion; I/R-Ins-before and I/R-IGF-1-before—insulin or IGF-1 was administered 1 h before the beginning of ischemic episode and then daily during 7 days of reperfusion; I/R-Ins-after and I/R-IGF-1-after—insulin or IGF-1 was administered 2 h after the end of ischemic episode and then daily during 7 days of reperfusion. (**A**) Representative photomicrographs of the CA1 region showing GFAP-immunopositive areas. Scale bar: 100 µm. (**B**) Quantification of the optical density of GFAP-immunopositive areas per unit of the hippocampus. Data are presented as medians (50% of the data) with interquartile ranges (Q1, Q3) (*n* = 3 animals per group, with at least 15 determinations per animal). Significance levels were determined using the Kruskal–Wallis test followed by post hoc analysis with Dunn’s test, and are indicated in the figure.

Similar to microglial activation, astroglial activation also contributes to the inflammatory response following cerebral ischemia–reperfusion, leading to astrogliosis. At 7 days after reperfusion, the relative optical density of GFAP-immunoreactive areas was three-fold higher in the I/R group than in the Sh-O group (0.0717 ± 0.0041 vs. 0.0232 ± 0.0017, *p* < 0.001), as determined by immunohistochemical analysis (Figure 12). Intranasal administration of either insulin or IGF-1—given before ischemia or 2 h after reperfusion onset and then daily for 7 days—effectively suppressed the development of astrogliosis. In all treatment groups, the differences from the I/R group were highly significant (*p* < 0.001).

**Figure 13:**
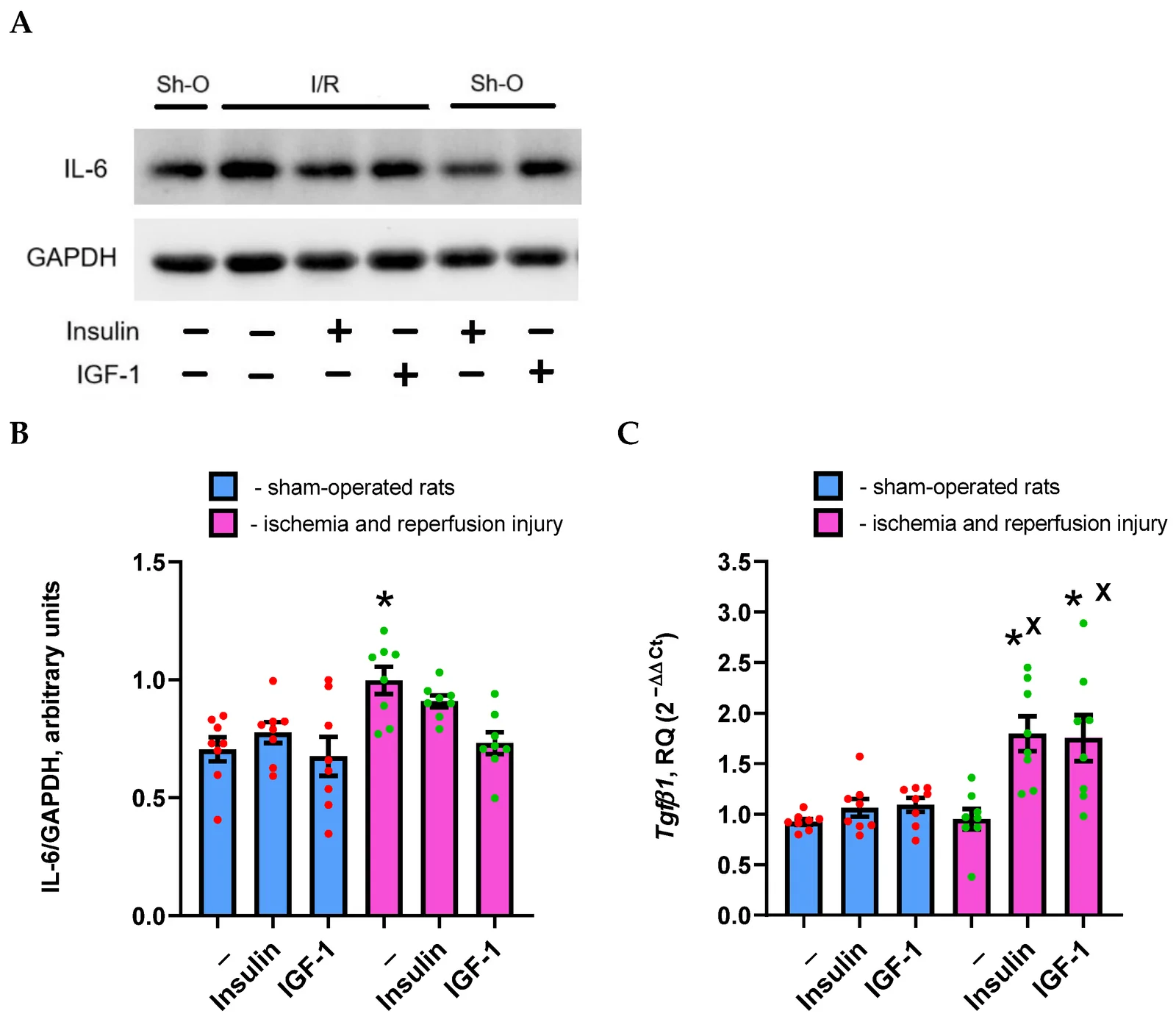
Effect of global forebrain ischemia and intranasal administration of insulin (0.6 IU/rat) and IGF-1 (0.6 µg/rat) on the expression of IL-6 and the *Tgfβ1* gene in the hippocampus of the rat brain. Insulin and IGF-1 were administered 1 h before the ischemic episode and then daily during 7 days of reperfusion. (**A**) Representative immunoblots. (**B**) Results of the IL-6/GAPDH ratio in the hippocampus of the rat brain. (**C**) Results of the *Tgfβ1* expression in the hippocampus of the rat brain. Data are presented as mean ± SEM (*n* = 8 animals/group). Statistical significance was assessed using one-way ANOVA followed by Tukey’s post hoc test for multiple comparisons: *—as compared to sham-operated rats, *p* < 0.05; X—as compared to rats subjected to ischemia and reperfusion, X—*p* < 0.05.

Activated microglia and astroglia serve as sources of both pro-inflammatory and anti-inflammatory cytokines. Western blot analysis of rat hippocampal lysates obtained after 7 days of reperfusion revealed a significant increase in the pro-inflammatory cytokine IL-6 in the I/R group compared with the sham-operated group (Figure 13A,B). Intranasal administration of insulin or IGF-1, given 1 h before ischemia and then daily throughout the 7-day reperfusion period, markedly reduced IL-6 levels in the hippocampus. These findings demonstrate that ischemia–reperfusion elevates IL-6 expression in the hippocampus and that both insulin and IGF-1 exert potent anti-inflammatory effects by suppressing this increase.

Real-time PCR analysis revealed that intranasal administration of insulin or IGF-1—given 1 h before ischemia and then daily during 7 days of reperfusion—significantly increased the expression of the anti-inflammatory cytokine gene *Tgfβ1* in the rat hippocampus. In contrast, ischemia–reperfusion alone did not induce any significant change in *Tgfβ1* expression during the same observation period (Figure 13C).

## 3. Discussion

The present study was designed to compare the neuroprotective and anti-inflammatory effects of intranasal insulin and IGF-1 in a rat model of transient global forebrain ischemia, with a particular focus on their ability to attenuate autophagic and apoptotic cell death in the hippocampal CA1 region. This investigation is based on our previous finding that intranasal bovine insulin at 0.5 IU per rat completely normalizes the ischemia-induced suppression of Na^+^, K^+^-ATPase activity, whereas a lower dose (0.25 IU) yields only partial restoration of this parameter [24].

A pronounced and significant neuroprotective effect was observed when either insulin (0.6 IU/rat) or IGF-1 (0.6 µg/rat) was administered intranasally 1 h prior to or 2 h following the ischemic insult, with subsequent daily administrations throughout the 7-day reperfusion period. In contrast, if the treatment was initiated 30 min post-ischemia, it failed to confer any protection. The doses of 0.6 IU insulin and 0.6 µg IGF-1 were chosen based on the standard, independently determined effective doses for each peptide in prior intranasal neuroprotection studies. Notably, our comparative analysis revealed that these specific dosages produced equipotent protective efficacy under the repeated administration regimen. Given that 1 IU of bovine insulin corresponds to 40 µg, the 0.6 IU dose equates to 24 µg per rat. Consequently, achieving comparable neuroprotection required a 40-fold higher mass of insulin relative to IGF-1. These findings indicate that, under conditions of transient global forebrain ischemia–reperfusion, hippocampal CA1 neurons exhibit a substantially higher sensitivity to IGF-1 than to insulin.

Having established the dose- and temporal dependency of this neuroprotection, we next investigated whether the daily administration of insulin and IGF-1 during 7-day reperfusion is essential for the observed efficacy. In a separate series of experiments, it was shown that a single intranasal administration of 0.6 IU insulin (administered either 1 h prior to ischemia or 2 h after it) or of 0.6 µg IGF-1 (given 2 h post-ischemia) failed to produce a significant increase in the level of viable CA1 neurons after 7 days of reperfusion (Table 1). At the same time, a single pre-ischemic dose of intranasal IGF-1 caused a moderate but statistically significant increase in neuronal survival. However, this value remained markedly lower than that achieved with the repeated additional daily administration of IGF-1 during first 7 days of reperfusion (*p* < 0.05, see Table 1). Thus, the data obtained indicate that the efficacy of intranasal insulin and IGF-1 can be augmented not only by dose increase, but also by repeated administration in the early reperfusion period. This observation may be of interest for the possible clinical use of insulin or IGF-1 in future as the drugs increase neuronal viability in cerebral ischemia–reperfusion injury. The principal difficulty in treating hemorrhagic stroke is achieving prompt medical intervention within the narrow therapeutic timeframe. By contrast, the repetitive administration of the agent later in the course presents negligible clinical hurdles. Our work substantiates the viability of this treatment regimen.

The effective neuroprotective doses of insulin observed in our study are consistent with those reported previously. For instance, intravenous or intraperitoneal administration of insulin at 0.3–1.0 IU/rat has been shown to confer neuroprotection in rat models of middle cerebral artery occlusion (MCAO) and transient global forebrain ischemia [52,53,54,55].

Unlike the consistent dosing profile of insulin, the effective dose of IGF-1 for neuroprotection and anti-inflammation is highly variable, depending largely on IGF-1 binding protein interactions, the ischemia–reperfusion model, animal age, and the route and timing of administration. Notably, intranasal delivery of ^125^I-IGF-I produces peak brain concentrations 12–20 times higher and more rapidly than other administration routes [56]. In MCAO models, the published effective dose range is accordingly wide [35,36,37,39,41,43]. While Schäbitz et al. [35] reported efficacy with continuous intracerebroventricular infusion of 33.3 µg/day for 3 days, Liu et al. [36,37] and Gong et al. [43] used substantially higher doses. However, some other studies have shown that much lower doses are also protective, especially with repeated administration, as in our own work. For example, Chang et al. [41] administered only 0.2 µg of IGF-1 per rat intramuscularly after MCAO and every other day during 7-day reperfusion thereafter, which significantly decreased apoptotic neuronal death and improved motor outcomes. Likewise, Rizk et al. [39] achieved significant reductions in lesion volume and apoptosis with a single dose of 1.45 µg/rat given either before or after ischemia.

We compared our results with those from the work of Lagina et al. [42], who used the same method of two-vessel forebrain ischemia with hypotension and subsequent reperfusion in rats. This study reported a statistically significant neuroprotective effect following intravenous administration of IGF-1 at 0.6 IU/kg. Given that 1 IU of IGF-1 is functionally equivalent to 1 µg (Medicines and Healthcare Products Regulatory Agency, 2025), the dose corresponded to 0.225 µg/rat. Using the intravenous injections, the authors observed a significant (though modest) increase in the level of viable neurons in the hippocampal CA1 region after 7 days of reperfusion; however, reducing the dose by half abolished this protective effect. In contrast, we administered a higher dose of IGF-1 (0.6 µg/rat) via the intranasal route, not only before and after the ischemic episode but also daily throughout the 7-day reperfusion period. This modified approach resulted in markedly enhanced neuroprotection, elevating the number of surviving CA1 neurons to levels indistinguishable from those in sham-operated controls.

It was of importance to consider the relative contributions of apoptosis and autophagy to CA1 neuronal death. The pivotal role of apoptosis in ischemic and reperfusion-induced neurodegeneration has been extensively documented, as well as the anti-apoptotic neuroprotective actions of insulin and IGF-1. However, while autophagy was long regarded as an exclusively homeostatic and protective pathway, it is now well recognized that its excessive activation following cerebral injury can culminate in autophagic cell death. Accordingly, the neuroprotective efficacy of numerous pharmacological agents against ischemia–reperfusion injury has been attributed to the concurrent suppression of both apoptosis and pathological activation of autophagy [57,58,59,60]. Conversely, recent reports have also demonstrated protective contexts in which autophagy activation mitigates ischemic brain damage [61,62,63]. Thus, in the setting of cerebral ischemia–reperfusion, autophagy exhibits a Janus-faced nature, with its net effect critically dependent on the magnitude of its activation. In some cases, excessive autophagy contributes to early neuronal injury during the acute ischemic phase and the initial reperfusion period; a more moderate autophagic activity during the later recovery phase may facilitate neuronal repair and resilience.

To directly investigate the contribution of these pathways, we administered the autophagy inhibitor 3-MA and the pan-caspase inhibitor Z-VAD-FMK intracerebroventricularly (ICV) prior to ischemia. Both significantly increased CA1 neuronal survival after 7 days of reperfusion (Figure 4). 3-MA administration reduced the death of neurons more than 50%; apoptosis inhibitor also markedly diminished neuronal death. These results provide evidence that excessive autophagy and apoptosis contribute to the neuronal death in this model. These data are in agreement with recent evidence that insulin administrations suppress not only apoptosis but also pathological autophagic activation following ischemia–reperfusion injury [64]. As far as IGF-1 is concerned, Thiebaut and co-authors [65] showed that thrombolysis by PLAT/tPA increases the level of serum-free IGF-1 leading to a decrease in deleterious autophagy activated as a result of ischemia and reperfusion. Collectively, these findings establish insulin and IGF-1 as neuroprotectors capable of mitigating the two principal modes of programmed cell death in the post-ischemic hippocampus.

To substantiate this functional protection, we examined the level of autophagy markers. Molecular profiling confirmed that both insulin and IGF-1 effectively prevented the pathologically elevated autophagic flux, increase in LC3B-II/ LC3B-I ratio, reversed ULK-1 phosphorylation dynamics (activation at Ser^555^ and deactivation at Ser^737^), caused by ischemia and reperfusion for 1 or 7 days. It was also found that insulin and IGF-1 upregulated *Mtor* expression—a key negative regulator of autophagy. These biochemical findings were further validated by reduced LC3B immunohistochemical staining in the CA1 region. Notably, across all assays, 0.6 IU insulin and 0.6 µg IGF-1 exhibited equivalent potency to inhibit autophagy. These data confirm that both peptides prevent pathological autophagy activation, thereby reducing autophagic neuronal death in the post-ischemic hippocampus.

Insulin and IGF-1 receptors are homologous disulfide-linked tetramers composed of extracellular ligand-binding α-subunits and transmembrane β-subunits with intrinsic tyrosine kinase activity [66]. The A- and B-isoforms of the insulin receptor bind insulin with high affinity but IGF-1 with low affinity [30,67]. Conversely, the IGF-1 receptor exhibits high affinity for both IGF-1 and IGF-2, while its affinity for insulin is 50-100-fold lower [20,30,68]. On a weight basis, 0.6 IU insulin (24 µg) exceeded the 0.6 µg IGF-1 dose by 40-fold. Accounting for molecular mass (bovine insulin, 5.73 kDa; rhIGF-1, 7.65 kDa), this corresponds to an approximately 53-fold molar excess of insulin relative to IGF-1. Notably, this ratio may be an underestimate, as administered IGF-1 is subject to sequestration by IGF-binding proteins in tissues, thereby reducing its free, bioactive concentration. We suggest that the equipotent neuroprotective actions of high-dose intranasal insulin and low-dose intranasal IGF-1 are attributable to insulin activation of not only insulin receptors, but IGF-1 receptors as well. In the literature there are such examples. Thus, in the work of Pires et al. [69], it was shown that systemic hyperinsulinemia suppressed autophagy in insulin receptor-deficient hearts via activation of IGF-1 receptors by insulin.

It should be noted that findings obtained from one model of ischemia–reperfusion injury may not be directly transferable to others. In our study, intranasal administration of 0.6 µg IGF-1 or 0.6 IU insulin, initiated 30 min after the ischemic episode and continued daily throughout 7 days of reperfusion, failed to protect CA1 hippocampal neurons in the transient global forebrain ischemia model. This contrasts with data from neonatal rats subjected to unilateral carotid artery ligation combined with hypoxia. In that model, intracerebroventricular IGF-1 was effective only when given within 1 h after the insult; administration prior to ischemia or later than 1 h after it yielded no benefit [34,40]. However, the same authors later reported a different pattern in adult rats exposed to the same hypoxic–ischemic injury. Guan et al. [38] found that a single ICV dose of 5 or 50 µg of rhIGF-1, administered 2 h after reperfusion, reduced cortical infarct size by more than 60%. Even at a lower dose (0.5 µg per adult rat), the infarction volume decreased by over 35%, although this effect did not reach statistical significance [38]. Taken together, these comparisons highlight that the protective efficacy and therapeutic window of IGF-1 and insulin are critically influenced by experimental conditions such as the specific model of ischemia used, animal age and the route of administration.

Neuronal viability depends on multiple factors, among which inflammation triggered by activated microglia appears to play a pivotal role in promoting neuronal death. Under physiological conditions, microglia serve not only as the brain’s primary innate immune cells but also actively contribute to the preservation of neuronal homeostasis. Their continuous crosstalk with neurons is essential for maintaining brain homeostasis, such processes as synapse formation, neuronal signal transmission, and cerebral metabolism [70,71].

Under pathological conditions, however, microglia shift toward a pro-inflammatory phenotype and initiate neuroinflammation. In the context of ischemic stroke, post-stroke neuroinflammation has recently attracted increasing research attention as a potential target for novel therapeutic strategies [72]. In this pathological state, microglial activation subsequently triggers astrocyte activation. Together, microglia and astrocytes are involved throughout all phases of ischemic stroke—from the early stage, where they mediate the initial wave of neuronal death, to the late stage, which encompasses phagocytic clearance and tissue repair [73].

The formation of reactive M1 microglia, which initiate neuroinflammation, is a hallmark not only of human ischemic stroke and MCAO models but also of global forebrain ischemia followed by reperfusion [74,75]. In this study, we aimed to evaluate whether insulin and IGF-1 could reduce the neuroinflammatory activation of M1 microglia in the CA1 region of the hippocampus following global forebrain ischemia and hypotension with subsequent reperfusion. Microglial activation is typically reflected by morphological changes, including process thickening and cell body hypertrophy—features that were observed in our ischemic animals. To quantify microglial activation, we performed immunohistochemical analysis of the level of Iba-1, a widely accepted marker that reliably labels microglia. Immunohistochemistry revealed a marked increase in activated microglia in the CA1 region after 7 days of reperfusion, as determined by elevated Iba-1 immunoreactivity and morphological features of microglia. Importantly, intranasal administration of either 0.6 IU insulin or 0.6 µg IGF-1, given before or 2 h after the ischemic episode and then daily throughout the 7-day reperfusion period, significantly reduced the microglial burden. In both experimental groups treated with these protectors, the number of microglia in the CA1 region was lowered to levels indistinguishable from those observed in sham-operated controls (Figure 11).

Astrocytes, like microglia, contribute to brain homeostasis by providing structural and nutritional support to neurons under physiological conditions [76]. Following stroke, however, activated microglia induce the formation of neurotoxic reactive astrocytes (A1 phenotype), which secrete IL-1β, IL-6, TNFα, and other cytotoxic factors [77]. Unlike their homeostatic counterparts, A1 astrocytes do not promote neuronal survival, growth, or synaptogenesis; instead, they actively induce the death of neurons and oligodendrocytes during the early days after ischemia and the beginning of reperfusion [73,77]. This creates a vicious cycle in the acute phase of ischemic stroke, wherein reciprocal activation of microglia and astrocytes further amplifies neuroinflammatory injury [73].

As a specific astrocytic marker, GFAP provides a reliable quantitative index of astrogliosis via its relative optical density. Using this measure, we found that cerebral ischemia–reperfusion elicited a pronounced astroglial response, whereas treatment with either insulin or IGF-1 effectively mitigated this activation, returning GFAP immunoreactivity to levels comparable to those seen in sham-operated animals.

The activation of microglia and astrocytes described above drives a cascade of neuroinflammatory events, characterized by the release of reactive oxygen species and pro-inflammatory cytokines [78,79,80]. In our model, transient global forebrain ischemia significantly elevated the hippocampal level of the pro-inflammatory cytokine IL-6 (Figure 13B). However, intranasal administration of either 0.6 µg IGF-1 or 0.6 IU insulin, given 1 h before the ischemic episode and then daily throughout the 7-day reperfusion period, markedly reduced IL-6 levels to values indistinguishable from those in sham-operated controls (Figure 13A,B). Real-time PCR analysis also revealed that the same intranasal pretreatment with IGF-1 or insulin significantly upregulated the expression of *Tgfβ1* (Figure 13C). Although reperfusion injury has been reported to activate TGF-β1 signaling, which is associated with extracellular matrix remodeling [81,82], transient global ischemia alone did not significantly alter *Tgfβ1* levels in our study. Post-ischemic upregulation of *Tgfβ1* may promote vascular remodeling, thereby preserving blood–brain barrier integrity [83,84]. Thus, IGF-1 and insulin may exert their neuroprotective actions not only by suppressing pro-inflammatory signals but also by modulating the level of multifaceted cytokine in the hippocampus of animals exposed to ischemia–reperfusion.

In this rat model, intranasal insulin (0.6 IU, administered 1 h before or 2 h after an ischemic episode and daily during reperfusion) affords neuroprotection and anti-inflammatory effects comparable to lower IGF-1 doses, markedly attenuating neuronal death in the hippocampal CA1 region by suppressing excessive autophagy and apoptosis. Crucially, insulin has a proven safety profile in humans, unlike IGF-1, whose clinical application is confirmed only for severe growth retardation caused by primary IGF-1 deficiency in children [45,47], due to the established neoplastic risk of its administration [22,23,44]. The intranasal route further offers a practical advantage for targeted delivery. Collectively, these findings position intranasal insulin as a promising agent for clinical use in ischemic/reperfusion brain injury.

## 4. Materials and Methods

### 4.1. The Design of Experiments and Animals Used

Experiments were carried out on adult male Wistar rats weighing 320–380 g. At the first stage of our study, 14 groups of rats were formed in order to compare the neuroprotective efficacy of intranasal insulin and insulin-like growth factor-1 (IGF-1) in a model of transient global brain ischemia followed by reperfusion. The animals were assigned to: group№ 1—Sham-operated rats (Sh-O); group № 2—Sh-O + 0.6 IU/rat of insulin (administration BEFORE the ischemic episode); group № 3—Sh-O + 0.6 IU/rat of insulin (administration AFTER the ischemic episode—in 30 min); group № 4—Sh-O + 0.6 IU/rat of insulin(administration AFTER ischemic episode—in 2 h); group № 5—Sh-O + 0.6 μg/rat of IGF-1 (administration BEFORE the ischemic episode); group № 6—Sh-O + 0.6 μg/rat of IGF-1 (administration AFTER the ischemic episode—in 30 min); group № 7—Sh-O + 0.6 μg/rat of IGF-1 (administration AFTER an ischemic episode—in 2 h); group № 8—rats with ischemia and reperfusion (I/R); group№ 9—I/R + 0.6 IU/rat of insulin (administration BEFORE an ischemic episode); group № 10—I/R + 0.6 IU/rat of insulin (administration AFTER an ischemic episode—in 30 min); group № 11—I/R + 0.6 IU/rat of insulin (administration AFTER an ischemic episode—in 2 h); group № 12—I/R + 0.6 μg/rat of IGF-1 (administration BEFORE an ischemic episode); group № 13—I/R + 0.6 μg/rat of IGF-1 (administration AFTER an ischemic episode—in 30 min); group № 14—I/R + 0.6 μg/rat of IGF-1 (administration AFTER an ischemic episode—in 2 h). In the case of using brains for fixation in paraformaldehyde in order to count the number of live neurons in the CA1 region of the hippocampus, and for immunohistochemistry, each group included 5–6 animals. The number of rats was increased to *n* = 8 in order to process hippocampus samples using immunoblotting and PCR.

In the second stage of the study, the animals underwent transient global brain ischemia followed by 7 days of reperfusion. To determine whether repetitive intranasal administrations of insulin or IGF-1 are necessary after carotid artery occlusion in combination with hypotension, these substances were administered once intranasally either 1 h before or 2 h after the ischemic episode. Since repeated intranasal injections of insulin and IGF-1 in Sh-O animals did not affect the number of live neurons in the CA1 region of the rat hippocampus, the study was limited to rats undergoing ischemia and reperfusion. The study involved six groups of animals: group № 1—Sham-operated rats (Sh-O); group № 2—rats with ischemia and reperfusion (I/R); group№ 3—I/R + 0.6 IU/rat of insulin (administration BEFORE an ischemic episode); group № 4—I/R + 0.6 IU/rat of insulin (administration AFTER an ischemic episode—in 2 h); group № 5—I/R + 0.6 μg/rat of IGF-1 (administration BEFORE an ischemic episode); group № 6—I/R + 0.6 μg/rat of IGF-1 (administration AFTER an ischemic episode—in 2 h). Each group consisted of 5-6 rats.

In the third stage of our research, we formed six groups to investigate the role of autophagy and apoptosis in the death of pyramidal neurons in the CA1 region of the hippocampus after ischemia/reperfusion injury. The inhibitor of autophagy 3-MA or inhibitor of apoptosis Z-VAD(OMe)-FMK were administered intracerebroventricularly (ICV) in the brain of rats. The experimental groups included: group № 1—Sham-operated rats (Sh-O); group № 2—Sh-O + 20 μg/rat of 3-MA (Sh-O + 3-MA); group № 3—Sh-O +10 μg/rat of Z-VAD(OMe)-FMK (Sh-O + Z-VAD-FMK); group № 4—rats with ischemia and reperfusion (I/R); group № 5—I/R + 20 μg/rat of 3-MA (I/R + 3-MA); group № 6—I/R +10 μg/rat of Z-VAD(OMe)-FMK (I/R + Z-VAD-FMK). Each group consisted of 5 rats.

### 4.2. Intranasal Administration of Insulin and IGF-1

Bovine insulin (#I5500, Sigma-Aldrich, St. Louis, MO, USA) at a dose of 0.6 IU per rat was administered intranasally. To do this, 12 μL of a solution containing 1 mg of insulin in 1 mL of citrate buffer was injected into each nostril of the animal. This corresponds to 0.6 international units (IU) of insulin. The citrate buffer was prepared by mixing equal volumes of 100 mM citric acid and 100 mM sodium citrate solutions, at a pH of 4.4.

Human recombinant IGF-1 (#CYT-216, ProSpec, Ness-Ziona, Israel) was used for intranasal injections of insulin-like growth factor-1. The peptide was dissolved in a sterile phosphate buffer (DPBS, pH 7.4). Rats were also injected with 12 µL in each nostril. A total of 24 μL contained 0.6 μg of IGF-1. Since rats can easily become accustomed to the procedure, the injections were administered without anesthesia. Sh-O rat received intranasally physiological solution. Insulin or IGF-1 was given intranasally 1 h before occlusion of the carotid arteries and hypotension, or 30 min or 2 h after an ischemic episode, and then daily for 1 or 7 days at the reperfusion stage.

### 4.3. Intracerebroventricular Injections of Apoptosis and Autophagy Inhibitors

In order to determine how the activation of autophagy and apoptosis affects the viability of neurons during two-vessel forebrain ischemia with hypotension and subsequent reperfusion, autophagy and apoptosis inhibitors (3-MA and Z-VAD-FMK, respectively) were administered intracerebroventricularly (ICV) into the lateral (I) ventricle in the brain of rats. In order to equalize the possible consequences of such administration, all rats in other groups of animals were injected ICV with sterile phosphate buffer, which was used to dissolve the inhibitors. After anesthetizing the rats with chloral hydrate (#C0073, TCI, Tokyo, Japan) administered intramuscularly at a dose of 400 mg/kg, the animals were placed in stereotaxis. For craniotomy, a dental bur was used in the area located above the first ventricle of the brain (coordinates: AP = −0.92 mm, L = 1.5mm, V = 3.5 mm relative to bregma) in accordance with the stereotactic atlas. Rats were injected ICV with 20 μg of 3-methyladenine (#M9281, Sigma-Aldrich, St. Louis, MO, USA) per rat, or 10 μg of Z-VAD(OMe)-FMK (#60332, Cell Signaling Technology, Danvers, MA, USA) per rat, dissolved in 4.5 μL of phosphate buffer, or a similar volume of phosphate buffer 1 h before ischemic exposure using a microsyringe (Hamilton, Reno, NV, USA).

### 4.4. Two-Vessel Forebrain Ischemia with Hypotension and Subsequent Reperfusion

The modern version of the method of two-vessel forebrain ischemia (as presented in [85]) was induced in rats by clamping the carotid arteries for 11.5 min in combination with lowering blood pressure to 50 mm Hg by drawing blood into a syringe with heparin. The femoral artery was catheterized with catheter (1.0 × 0.6 × 210 mm, 23G) purchased from SciCat (Balashiha, Russia). Forebrain reperfusion was carried out by decompressing the carotid arteries and returning blood with heparin, taken with a syringe at the stage of ischemia, into the blood stream. Sham-operated rats were used as control animals. After completion of ischemia, the rats had their wounds treated with streptocide powder. The surgical incisions were sutured with sterile non-absorbable lavsan threads (MP1.5 UP4-0, Lintex LLC, Saint-Petersburg, Russia). Postoperative care of the rats was carried out for 1 or 7 days while keeping them on a standard diet. The animals were housed in cages (5 rats/cage) under standard environmental conditions (12:12 h light–dark cycle; ambient temperature 23 ± 2 °C; food and water available ad libitum).

### 4.5. Counting the Number of Live Neurons in the CA1 Region of the Rat Hippocampus by Modified Nissl Method

Animals anesthetized with chloral hydrate were decapitated in 7 days after the start of reperfusion. The brains were excised, washed with chilled physiological solution and placed for 7 days in 4% paraformaldehyde in 10 mM phosphate buffer (+4 °C). For cryoprotection, the brain was kept in a solution of 30% sucrose in 20 mM PBS for 7 days at +4 °C, after which it was frozen in isopentane and then stored at −80 °. Alternating series of frontal slices (10 μm) were obtained from rat hippocampus using a cryostat (Leika Biosystems, Nussloch, Germany). Every 10-th slice was mounted on gelatin-coated glass (BioVitrum, Saint-Petersburg, Russia). To assess the number of live neurons, toluidine blue staining was performed using the modified Nissl method. Each group of animals included 5–6 rats. Images were taken using a Carl Ziess Axio A1 microscope (Zeiss, Oberkochen, Germany) with a built-in AxioCam 712 color television camera (Zeiss, Oberkochen, Germany) and the Zen 3.4 Program (Zen pro). The number of viable cells in the hippocampal CA1 was counted over the region length of 300 μm, using Bio7 software (Bielefeld, Germany). The total number of measurements of live neurons for each animal was not less than 30.

### 4.6. Immunohistochemical Analysis of Protein Expression in Brain Slices

Coronal brain slices (15 μm thickness) containing the dorsal hippocampus were mounted onto Superfrost^®^ Plus adhesion microscope slides (Menzel Gläser, Braunschweig, Germany) in an alternating pattern (every fifth slide) and stored at –20 °C until use. Prior to the immunohistochemical procedure, slides were allowed to dry at room temperature for 24 h. For antigen retrieval, slides were first rinsed in sodium phosphate-buffered saline (PBS, pH 7.4), then transferred to citrate buffer (pH 6.0) and heated in a closed container by boiling for 3 min. After cooling to room temperature in air, slides were washed three times in PBS for a total of 10 min. Endogenous peroxidase activity was blocked by incubating the sections in 0.6% hydrogen peroxide (diluted in PBS) for 30 min at room temperature. Following three washes in PBST (PBS containing 0.1% Triton X-100) for 15 min, non-specific binding sites were blocked by incubation in a 5% blocking solution (2% horse serum, 3% goat serum in PBST) for 1 h at room temperature. Sections were then incubated overnight (12 h) at room temperature with the following primary antibodies diluted in 1% blocking solution (1% horse and goat serum in PBST): rabbit anti-Iba1 (1:500, #NBP2-19019, Novus Biologicals, Centennial, CO, USA), mouse anti-GFAP (1:1500, provided by the Federal Center for Brain and Neurotechnology, Moscow, Russia), and rabbit anti-LC3B (1:200, #83506, Cell Signaling Technology, Danvers, MA, USA). After thorough washing in PBST (three changes, 10 min each), sections were incubated for 1 h at room temperature with biotinylated secondary antibodies (goat anti-rabbit and goat anti-mouse, Vector Laboratories, Burlingame, CA, USA) diluted 1:800 in PBST. Following additional washes (three times in PBST, 10 min each), the sections were treated with streptavidin–peroxidase complex (Sigma, Saint Louis, MO USA) diluted 1:1000 in PBS for 1 h at room temperature. The peroxidase reaction was visualized using a freshly prepared solution containing 0.05% 3,3′-diaminobenzidine (DAB) and 0.015% hydrogen peroxide in PBS. The reaction was monitored under a light microscope and stopped by washing in distilled water. Finally, sections were dehydrated through a graded ethanol series (70%, 95%, and 100%), cleared in ortho-xylene, and mounted under 0.2 mm thick coverslips using mounting resin Vitrogel (Biovitrum, Saint Petersburg, Russia). Images of the hippocampal CA1 region were acquired using a Carl Zeiss Axio A1 microscope (Zeiss, Oberkochen, Germany). Quantitative assessment of immunopositive cells was performed using Bio7 software (Bielefeld, Germany). Immunoreactive signal intensities for LC3B and GFAP were evaluated and expressed as relative optical density (ROD) units. For LC3B immunoreactivity in CA1 pyramidal neurons, the soma of each selected cell was manually outlined. The optical density reflecting the content of an LC3B-immunopositive substance was calculated as the difference between intensely colored neurons containing an immunoreactive substance and the intensity of background coloring (not containing an immunoreactive substance) on the same section. The background value was sampled from an adjacent field within the same microscopic frame, ensuring the sampled area was entirely devoid of large pyramidal somata. ROD = log_10_(I_bg/I_cell), where I_bg is the mean background gray value and I_cell is the mean gray value of the outlined soma. For GFAP immunoreactivity, the intensity of the immunopositive area was quantified within the CA1 region. The area was manually outlined. The final value was corrected by subtracting the area occupied by nonspecific staining measured in an adjacent extra-hippocampal field. For Iba-1 immunostaining, microglial cells with hypertrophied body and thick processes were counted manually. The number of Iba-1-positive activated cells was calculated and expressed as the mean number of labeled cells per unit area corresponding to 300 µm length of the hippocampal CA1 region.

### 4.7. Analysis of Gene Expression by Reverse Transcription Polymerase Chain Reaction (RT-PCR)

Total RNA was extracted from hippocampal samples using the RNA Extract reagent (Eurogen, Moscow, Russia) according to the manufacturer’s instructions. RNA concentration was measured with a NanoPhotometer N60 spectrophotometer (Implen, Munich, Germany). Purity was assessed by the absorbance ratio at 260 nm and 280 nm; only samples with a ratio between 1.9 and 2.2 were used for further analysis. Complementary DNA (cDNA) was synthesized using the MMLV RT Kit (Eurogen, Russia) with random hexamer primers and 1 µg of total RNA per reaction, following the manufacturer’s protocol. Each amplification reaction was performed in a total volume of 25 µL containing 10 ng of cDNA, 0.4 µM of each gene-specific forward and reverse primer, and qPCR mix-HS SYBR + LowROX reagent (Eurogen, Moscow, Russia). The reaction was run on a CFX96 real-time PCR detection system (Bio-Rad, Hercules, CA, USA). The expression of target genes encoding the proteins of interest and reference genes (*18S rRNA* and *Actin B*) was determined using the following primer sequences: *Tgfb1*—GACTCTCCACCTGCAAGACC (For) and GGACTGGCGAGCCTTAGTTT (Rev); *Mtor*—GGGCAGCAACAGTGAAAGTG (For) and ACGGAAGAAGCCTTGGACAG (Rev); *18S rRNA*—GGACACGGACAGGATTGACA (For) and ACCCACGGAATCGAGAAAGA (Rev); *Actin B*—GCGAGTACAACCTTCTTGCAG (For) and CTGACCCATACCCACCATCAC (Rev).

Results were analyzed using Bio-Rad CFX Manager 3.1 software. For normalization, the ΔCt index was calculated as the difference between the geometric mean of the Ct values of the reference genes (*18S* and *Actin B*) and the Ct value of the target transcript. Relative expression levels of the target genes were assessed using the ΔΔCt method, where ΔΔCt = ΔCt(control sample)—ΔCt(test sample). All data are presented as relative quantification (RQ) values of target gene mRNA levels, normalized to the Sh-O group.

### 4.8. Caspase-3 Activity Assay

Hippocampal tissue samples were homogenized in 200 µL of ice-cold lysis buffer consisting of 5 mM CHAPS, 5 mM dithiothreitol (DTT), and 50 mM HEPES (pH 7.4). The homogenates were incubated on ice for 30 min to facilitate cell lysis, followed by centrifugation at 1000× *g* for 5 min at 4 °C. The resulting supernatants were collected as cell lysates for subsequent assays. Caspase-3 activity was measured using a colorimetric assay based on the cleavage of the specific substrate Ac-DEVD-pNA (N-acetyl-Asp-Glu-Val-Asp-p-nitroanilide, #A860497, Macklin, Shanghai, China). The reaction mixture (total volume 100 µL) was prepared in 96-well plates and contained the following components: reaction buffer (20 mM HEPES, 0.1% CHAPS, 2 mM EDTA, and 5 mM DTT), 0.2 mM Ac-DEVD-pNA substrate, and an aliquot of cell lysate. The amount of protein from the lysate added to each well was standardized within the range of 50–200 µg per reaction, based on protein concentration determined by the Lowry method. For each sample, reactions were set up in duplicate parallel wells. Plates were incubated at 37 °C for 2 h, during which enzymatic cleavage of the substrate released p-nitroanilide (pNA). The absorbance of pNA was measured at 405 nm using a CLARIOstar multimodal plate reader (BMG Labtech, Ortenberg, Germany). Caspase-3 activity in each sample was calculated from the absorbance values and normalized to the total protein content of the corresponding lysate, as determined by the Lowry method.

### 4.9. Immunoblotting

Hippocampal samples were homogenized in lysing buffer at a ratio of 1:20 (*w*/*v*). The buffer contained 20 mM Tris-HCl (pH 7.5), 150 mM NaCl, 2 mM EGTA, 2 mM EDTA, 0.5% sodium deoxycholate, 0.5% Triton X-100, 15 mM NaF, 10 mM sodium glycerophosphate, 10 mM sodium pyrophosphate, 1 mM Na_3_VO_4_, 1 mM PMSF, 0.02% NaN_3_, and a protease inhibitor cocktail (BioinnLabs, Rostov-on-Don, Russia). The homogenate was centrifuged at 1000× *g* for 10 min at 4 °C to pellet intact cells and nuclei. The protein concentration in the resulting supernatant was determined using a modified Lowry method. Aliquots of hippocampal lysates containing 20 µg of total protein per lane were separated by sodium dodecyl sulfate-polyacrylamide gel electrophoresis (SDS-PAGE) on 9–13% polyacrylamide gels according to Laemmli’s method. Proteins were then transferred onto polyvinylidene difluoride (PVDF) membranes (0.2 µm pore size, Bio-Rad, USA) or nitrocellulose membranes (0.45 µm, GE Healthcare, UK). Non-specific binding was blocked by incubating the membranes with 5% (*w*/*v*) non-fat dry milk in Tris-buffered saline containing 0.1% Tween-20 (TBST) for 1 h at room temperature. Membranes were incubated overnight at 4 °C with primary antibodies diluted in TBST containing 5% BSA or 5% milk according to the manufacturer’s recommendations. The following primary antibodies were used: anti-LC3B-I/II (1:1000, #2775, Cell Signaling, Danvers, MA, USA), anti-IL-6 (1:1000, #DF6087, Affinity Biosciences, Changzhou, China), anti-pULK-1 (Ser^555^) (1:1000, #5869, Cell Signaling, USA), anti-pULK-1 (Ser^757^) (1:1000, #14202, Cell Signaling, USA), anti-ULK-1 (1:1000, #8054, Cell Signaling, USA), and anti-GAPDH (1:3000, #T0004, Affinity Biosciences, China). After three washes with TBST (5 min each), membranes were incubated with horseradish peroxidase (HRP)-conjugated anti-mouse or anti-rabbit secondary antibodies (Cell Signaling, USA) for 1 h at room temperature. Following additional washes, the chemiluminescent signal was developed using a Clarity Western ECL kit (Bio-Rad, USA). Chemiluminescence images were captured using a ChemiDoc gel imaging system (Bio-Rad, USA). Densitometric analysis of protein bands was performed using Bio-7 software. Normalization was carried out against total ULK-1 (for phosphorylated ULK-1) or GAPDH (for LC3B-II and IL-6).

### 4.10. Statistical Analysis

Statistical analysis was performed using GraphPad Prism 8.4.3 (GraphPad Software, San Diego, CA, USA). Data normality was assessed using the Kolmogorov–Smirnov test. For data following a normal distribution, parametric tests were applied: comparisons between two groups were conducted using Student’s unpaired *t*-test, while comparisons among three or more groups were performed using one-way analysis of variance (ANOVA), followed by appropriate post hoc tests. Results are presented as mean ± standard error of the mean (M ± SEM). Statistical significance was set at *p* < 0.05. For data that did not meet the normality assumption, non-parametric analyses were employed. Differences among groups were evaluated using the Kruskal–Wallis test, followed by Dunn’s post hoc test for pairwise comparisons. Data are presented as the median (M) with interquartile ranges (Q1; Q3). Differences were considered statistically significant at *p* < 0.05.

## 5. Conclusions

The present study demonstrates that intranasal administration of either insulin or IGF-1 provides robust neuroprotection against delayed neuronal death in the hippocampal CA1 region following transient global forebrain ischemia and reperfusion. Both compounds significantly increased neuronal survival when administered either 1 h before or 2 h after the ischemic insult, followed by daily dosing throughout the 7-day reperfusion period. Notably, no significant differences in neuroprotective efficacy were observed between insulin (0.6 IU/rat) and IGF-1 (0.6 µg/rat) under any administration schedule.

A critical finding of this study is the importance of administration timing. Neither insulin no IGF-1 exerted protective effects when first administered 30 min after reperfusion onset, suggesting that the therapeutic window for intranasal delivery in this model opens after this time point. In addition, single-dose administration was largely ineffective, whereas additional daily dosing throughout reperfusion produced marked neuroprotection. These results indicate that both the timing of the initial dose and continued daily administration during the first days of reperfusion are essential for achieving maximal neuroprotection.

We found that excessive autophagy and apoptosis represent the main pathways of neuronal death in the hippocampal CA1 region following ischemia–reperfusion. Inhibition of autophagy with 3-MA or apoptosis with Z-VAD-FMK significantly increased neuronal survival, rescuing more than 50% and 40% of neurons from death, respectively. Importantly, both insulin and IGF-1 suppressed ischemia–reperfusion-induced autophagy, as evidenced by reduced LC3B-II levels, decreased pULK-1(Ser^555^)/ULK-1 ratio, and increased mTOR gene expression. Both compounds also markedly reduced caspase-3 activity, confirming potent anti-apoptotic effects.

Furthermore, insulin and IGF-1 exerted significant anti-inflammatory effects. Both compounds reduced the number of activated Iba-1-positive microglia with thickened processes and hyperthrophy of cell bodies, which was increased by ischemia–reperfusion, and suppressed astrogliosis (GFAP expression) in the CA1 region. These effects were accompanied by decreased pro-inflammatory IL-6 levels and increased anti-inflammatory *TGFβ1* expression.

Notably, the molar dose of insulin required to achieve comparable neuroprotection with IGF-1 was approximately 53 times higher than that of IGF-1. Taking into account that CA1 region expresses both insulin and IGF-1 receptors, and that high-concentration of insulin can cross-activate IGF-1 receptors, the neuroprotective effects of insulin at the dose used may be mediated at least in part through activation of IGF-1 receptor signaling.

As for the effect of IGF-1, the serious concerns have arisen regarding its possible stimulation of benign and malignant neoplasms formation in people to whom IGF-1 was administered. Taking into account the oncogenic effects of IGF-1, insulin appears to be better candidate for use in clinics.

## Figures and Tables

**Table 1:** Comparison of neuroprotective effects of single versus repeated intranasal insulin (0.6 IU/rat) or IGF-1 (0.6 µg/rat) administered before or after transient forebrain ischemia in rats. Outcome: number of live neurons in hippocampal CA1 region at 7 days of reperfusion.

Insulin or IGF-1 Were Administered One Time (1 h Before or 2 h After the Ischemic Episode)		Insulin or IGF-1 Were Administered 1 h Before or 2 h After the Ischemic Episode and Then DAILY During 7 Days of Reperfusion	
Sham-operated rats (Sh-O)	61.04 ± 1.49	Sham-operated rats (Sh-O)	61.04 ± 1.49
Ischemia and reperfusion (I/R)	18.20 ± 1.73 ***	Ischemia and reperfusion (I/R)	18.20 ± 1.73 ***
I/R + Insulin, before	36.53 ± 4.88 **	I/R + Insulin, before	43.41 ± 7.69 ^X^
I/R + Insulin, after	37.43 ± 5.5 *	I/R + Insulin, after	46.04 ± 7.31 ^X^
I/R + IGF-1, before	40.83 ± 4.52 *^X#^	I/R + IGF-1, before	55.76 ± 2.87 ^XX^
I/R + IGF-1, after	36.18 ± 10.68 **	I/R + IGF-1, after	52.7 ± 1.67 ^XX^

Abbreviations used: IGF-1—insulin-like growth factor-1, Sh-O—sham-operated rats, I/R—rats subjected to ischemia and reperfusion. The transient global ischemia of rat forebrain was used. Insulin and IGF-1 were administered 1 h before or 2 h after the ischemic episode. In some experiments such administration was accompanied by administration of these protectors daily during 7 days of reperfusion. Results of quantitative analysis of live neurons in a linear 300 µm segment of the CA1 pyramidal cell layer after Nissl staining are presented as mean ± SEM (*n* = 4–6 rats per group; for each rat, at least 30 determinations were performed). Statistical significance was assessed using one-way ANOVA followed by Tukey’s post hoc test for multiple comparisons: *, ** and ***—as compared to sham-operated rats, * *p* < 0.05, ** *p* < 0.01, *** *p* < 0.001; ^X^ and ^XX^—as compared to ischemic and reperfused rats, ^X^—*p* < 0.05, ^XX^—*p* < 0.001. The difference is significant by Student’s *t* test: #—as compared to rats to whom IGF-1 was administered not only before ischemia, but also daily during 7 days of reperfusion, *p* < 0.05.

## Data Availability

The original contributions presented in this study are included in the article. Further inquiries can be directed to the corresponding author.

## Abbreviations

The following abbreviations are used in this manuscript:

ICV	Intracerebroventriculy
IHC	Immunohistochemical
IGF-1	Insulin-like growth factor-1
I/R	Rats subjected to ischemia and reperfusion
IU	International units
MCAO	Middle cerebral artery occlusion
Sh-O	Sham-operated rats
3-MA	3-methyladenine
Z-VAD-FMK	Carbobenzoxy-valyl-alanyl-aspartyl-[O-methyl]-fluoromethylketone
